# Genomic personalities of *Dehalococcoides* subspecies and *Dehalogenimonas* illuminate complete trichloroethene dechlorination in high-salt conditions

**DOI:** 10.1093/ismeco/ycaf101

**Published:** 2025-06-19

**Authors:** Wei-Yu Chen, Yun-Chi Lan, Jiung-Wen Chen, Jer-Horng Wu

**Affiliations:** Department of Environmental Engineering, National Cheng Kung University, Tainan City 70101, Taiwan; Department of Environmental Engineering, National Cheng Kung University, Tainan City 70101, Taiwan; Department of Biology, University of Alabama at Birmingham, Birmingham, AL 35294, United States; Department of Environmental Engineering, National Cheng Kung University, Tainan City 70101, Taiwan

**Keywords:** Salt tolerance, Trichloroethene dechlorination, Dehalococcoides, Dehalogenimonas, Ribosomal protein L33p gene

## Abstract

Global salinization increasingly threatens ecosystem integrity and the regulation of biogeochemical cycles. Our study reveals novel insights into the microbial contributions to the organohalide decomposition in saline environments, demonstrating the unprecedented ability of organohalide-respiring bacteria *Dehalococcoides* and *Dehalogenimonas* to completely dechlorinate trichloroethene to non-toxic ethene under hypersaline conditions (up to 31.3 g/L) in long-term operations. Using gradient salinity reactors and metagenomic analyses, we identified the evolved genomic features associated with high-salt tolerance. The Cornell subgroup of *Dehalococcoides* and *Dehalogenimonas* exhibit significantly lower average protein isoelectric points and retain the ribosomal protein L33p gene, unlike the Victoria and Pinellas subgroups. *Dehalococcoides* shows subspecies-level genomic divergence and unique codon usage biases. Intriguingly, the L33p gene is found in diverse bacterial phyla from saline environments, suggesting it may provide a growth advantage under salt stress. These genomic traits, hypothesized to enhance salt tolerance and dechlorination efficiency under salt stress, correlate with performance at elevated salinity. Our findings advance the understanding of microbial salt adaptation mechanisms and support the development of bioremediation strategies tailored for saline environments.

## Introduction

Salinization is a long-standing problem in coastal areas and has emerged as a major challenge worldwide, further exacerbated by climate change [1]. In soils and groundwater, prolonged drought, seawater intrusion, rising sea levels, and anthropogenic activities lead to high salt levels [2–4]. This salinization crisis adversely affects agricultural productivity, reduces biodiversity, contaminates freshwater resources [5], and disrupts the microbial communities that are essential for maintaining biogeochemical cycles [5–7].

Organohalide-respiring bacteria (OHRB) mediate the process of organohalide respiration with their distinctive reductive dehalogenase (RDase) enzymes, which release halogen ions from the carbon–halogen bonds of organohalides. These abilities enable OHRB to contribute to halogen cycling on Earth. Among the diversified OHRB, only *Dehalococcoides* and *Dehalogenimonas* can produce ethene from the process of reductive trichloroethene (TCE) dechlorination and form a terrestrial cluster branching from marine *Dehalococcoidia* clusters in the 16S rRNA phylogenetic tree [8]. *Dehalococcoides mccartyi (Dhc. mccartyi)*, extensively studied since the 1990s, is central to biotechnological remediation of chlorinated solvents [9, 10]. Depending on their 16S rRNA gene sequence similarity, *Dhc. mccartyi* can be divided into three subgroups: Cornell, Pinellas, and Victoria [11]. The reductive dechlorination of TCE to ethene is strain-dependent but is not associated with their distribution in the subgroups, with many strains dechlorinating TCE and dichloroethene (DCE) but only a few capable of dechlorinating vinyl chloride (VC) to ethene, a nontoxic product, to achieve detoxification.

*Dhc. mccartyi* exhibit varying degrees of tolerance to salinity, with tolerable salinity levels ranging from 3 to 15 g/L in strains within the Victoria (strains UCH007 and CG1) and Pinellas (strain NIT01) subgroups [12–14]. Recent studies have shown that *Dehalogenimonas etheniformans* GP can completely dechlorinate chloroethene to ethene [15, 16]. However, its dechlorination activity considerably decreases at salinity levels exceeding 7.5 g/L [15]. In another study, no ethene was produced during the reductive dechlorination of tetrachloroethene (PCE) at salinity levels of 2.5–50 g/L [14]. Few studies have reported the growth of *Dehalococcoides* or the complete dechlorination of TCE to ethene by *Dehalococcoides* or *Dehalogenimonas* spp. at salinity levels over 20 g/L. However, a recently isolated *Dehalogenimonas* strain from estuarine sediments was shown to dechlorinate 1,2-dichloroethane to ethene under saline conditions, expanding our knowledge of OHRB activity in marine environments [17]. Despite these advances, the molecular mechanisms enabling *Dehalogenimonas* and *Dehalococcoides* to perform complete TCE dechlorination under high-salinity conditions remain largely unknown. This gap in the literature presents a major challenge for the remediation of contaminated sites in high-salinity environments.

Salinity is a key determinant of microbial distribution, distinguishing freshwater from marine communities. High salinity inhibits the growth of freshwater microorganisms through multiple mechanisms, such as cytoplasmic dehydration and disruption of membrane integrity, and impaired enzymatic function [18]. Salt stress also increases energy expenditure, as cells must actively maintain ion gradients to preserve homeostasis, diverting resources from growth and metabolism [19]. To cope with osmotic stress, microorganisms can utilize various osmoregulatory strategies. These include the accumulation of compatible organic solutes (osmolytes) through de novo synthesis and transport, adjustment of membrane lipid structure, and the salt-in mechanism, which involves the active transport of potassium ions to offset external ionic strength [20].

Salt-tolerant microorganisms have evolved complex molecular mechanisms to maintain cellular homeostasis and combat salt stress. These include regulatory networks that control osmotic stress response pathways [20–22]. Enhanced translation efficacy can be achieved by modifying tRNA anticodon interactions and codon usage preferences, which influence protein folding, stability, and functionality [23, 24]. Under stress, expression of ribosomal protein genes is tightly regulated to optimize ribosome assembly, stability, and performance [25–27]. Additionally, halophilic microorganisms typically possess proteomes with lower average isoelectric points (pIs) [28, 29]. Despite these understandings, the genomic characteristics of biogeochemically important OHRB, particularly *Dehalococcoides* and *Dehalogenimonas* spp., remain poorly characterized in the context of salt stress.

In this study, we used batch experiments and long-term bioreactors with varying degrees of salinity to investigate physiological responses of *Dehalococcoides* and *Dehalogenimonas* spp. in the consortia to high-salt conditions. Comparative genome analysis was performed to elucidate the molecular traits evolved in these species, including osmotic regulation mechanisms, translational machinery adjustments, and protein features that contribute to salt tolerance. Our findings advance a deep understanding of organohalide respiration in saline sites and offer novel insights for optimizing bioremediation approaches in salt-impacted ecosystems.

## Materials and methods

### Batch test setup and chemical analysis

Anaerobic batch experiments were conducted using an enrichment culture from a long-term TCE-dechlorinating reactor over nearly 2000 days of operation. This inoculum culture was originally established at 3.5 g/L NaCl salinity and gradually adapted to around 7.62 g/L NaCl, maintaining *Dehalococcoides* and *Dehalogenimonas* as dominant OHRB [30]. Triplicate bottles were prepared with salinity levels of 5, 10, 15, and 20 g/L NaCl. Each 125-ml bottle contained 45 ml anaerobic medium [31] and 5 ml enrichment culture, anaerobically inoculated and sealed under oxygen-free dinitrogen. Subsequently, 0.5 ml of vitamin mixture [32] was added to each bottle (vitamin B12 final concentration: 10 μg/L). A final concentration of 0.268 mM TCE in methanol was added, with methanol and lactate as electron donors. To validate the enhanced salt tolerance observed in the reactor experiments during long-term operation, additional batch tests were conducted using 10% (v/v) inoculum from the saline reactor. Following the same protocol, these tests were performed at 21 g/L NaCl with an initial TCE dosage of 0.07 mmol/bottle. Chloroethene and methane concentrations were quantified using gas chromatography with flame ionization detection, as described in previous publications [32, 33].

### Setup and operation of fed-batch reactors

Three parallel fed-batch reactors were operated to evaluate TCE detoxification under varying salinity conditions: one with gradually decreasing salinity (SD reactor), one with gradually increasing salinity (SI reactor), and one with sharply increasing salinity (SM reactor) (Supplementary Table 1). Each reactor had a working volume of 1.8 L, consisting of 1.4 L anaerobic medium containing resazurin sodium (0.5 mg/L) as a redox indicator [32] and 0.4 L of the inoculum culture described in Section 2.1, and stirred at 60 rpm. Vitamin B12 was supplemented at a final concentration of 10 μg/L on days 0, 31, 161, and 202. The reactors were operated in seven stages, each with a specific salinity level. The fed-batch mode operated recurrently, cycling through feeding and reaction steps. Two to four cycles were performed per stage to ensure data reproducibility. To adjust salinity, 200 ml of the mixed liquid (approximately 11.2% of the total reactor volume) was replaced with an anaerobic medium containing different NaCl concentrations using a peristaltic pump (BT100L; Lead Fluid, Hebei, China). The discharged liquid (200 ml) was filtered using a 0.2-μm membrane and analyzed for water quality and microbiome. TCE (dissolved in 50 μl methanol), lactate (360 μl), or emulsified oil substrate (EOS) (soybean oil (49% v/v) and Tween 80 (2%, v/v) in water [33]) (960 μl), was manually injected into the reactors via syringe. The medium was buffered using bicarbonate (1.5 g/L) and adjusted to pH 6.8–7.0 with hydrochloric acid (5 N). Throughout the experiment, the pH (7.1 ± 0.06) and temperature (29.8 ± 0.3°C) of the reactors were monitored through an online probing system (PC-3110; Suntex, Kaohsiung, Taiwan). Headspace gas samples were regularly collected for analyzing chloroethenes, ethene, and methane. First-order kinetics was used to determine the dechlorination rate constant (*k*_TCE-to-ETH_) based on chlorine equivalents of chloroethenes (TCE: 3, DCE: 2, VC: 1), as previously described [30]. Chloride and sodium ions were analyzed using ion chromatography.

### Nucleic acid extraction, cDNA synthesis, and quantitative PCR

DNA and RNA were extracted from sludge samples using DNeasy and RNeasy PowerWater kits (Qiagen). After DNase treatment, RNA was converted to cDNA. qPCR was performed targeting 16S rRNA, *tceA*, and *vcrA* genes of *Dehalococcoides* and *Dehalogenimonas* [30]*.*

### Full-length 16S rRNA gene amplicon sequencing and analysis

Full-length 16S rRNA gene amplicon libraries were sequenced using PacBio Sequel II technology (BIOTOOLS, New Taipei City, Taiwan). The bacterial 16S rRNA genes were PCR-amplified using barcoded primers: Forward (5’Phos/GCATC-[16-base barcode]-AGRGTTYGATYMTGGCTCAG-3′) and Reverse (5’Phos/GCATC-[16-base barcode]-RGYTACCTTGTTACGACTT-3′). Circular consensus sequences were generated with stringent quality control (minimum passes ≥5, predicted accuracy ≥0.999). Sequences were processed using QIIME2 (v.2022.2) with DADA2 algorithm for denoising and chimera removal. On average, 90.7% ± 3.7% sequences passed quality filtering, generating 1438 amplicon sequence variants (mean length 1457 bp) across 23 samples. Taxonomic assignment and phylogenetic analysis followed previously described methods [30]. Sequence data were deposited in NCBI’s Sequence Read Archive under BioProject PRJNA1121973 (accession numbers SRR29729084-SRR29729104).

### Shotgun metagenomic sequencing and bioinformatic analyses

#### Sequencing, genome assembly, and phylogenomic analysis

DNA samples from three reactor stages (SI reactor at 14.8 g/L salinity; SM reactor at 17.8 g/L and 31.3 g/L salinity) were sequenced using Illumina HiSeq platform (BIOTOOLS, New Taipei City, Taiwan). After quality control and trimming, clean sequences were assembled using MEGAHIT [34] in meta-sensitive mode. Metagenome-assembled genomes (MAGs) were obtained through VAMB [35] binning. Taxonomic classification was performed using GTDB-TK [36]. Reference genomes of *Dehalococcoides* and *Dehalogenimonas*, along with metagenomic data from a previous study on TCE dechlorination under high-salinity conditions [14], were obtained from NCBI genome database (March 2024). A phylogenomic tree was constructed using GToTree [37] based on single-copy universal marker genes.

#### Functional annotation and comparative analyses

Open reading frames prediction was performed using Bakta (v.1.8.2) [38], with validation through RAST server [39]. Compatible solute-related genes were identified through EggNOG-mapper annotation against the EggNOG 5.0 reference database. Average Nucleotide Identity (ANI) values between genomes were calculated using FastANI [40] to establish taxonomic relationships and strain-level distinctions. Protein pI profiles were calculated using EMBOSS pI calculator [41] to identify acidic proteins associated with halophilic adaptation. Codon adaptation index and codon usage patterns were analyzed using CodonW (v.1.4.2) and EMBOSS CUSP program [41].

#### Genomic context and abundance analysis

rRNA gene locations were annotated using Bakta and visualized with ChiPlot (https://www.chiplot.online/). The ribosomal gene regions were analyzed using OperonMapper [42] and Clinker & Clustermap.js [43]. The gene abundance (L33p and L34p) was determined by functional annotation with EggNOG-mapper (v.2.1.9) using KEGG orthology K02913 and K02914, with relative abundance calculated as the ratio of ribosomal gene copies to total predicted ORFs. Gene co-occurrence patterns were analyzed using STRING database (v12.0) [44]. Metagenomic data were deposited in NCBI’s Sequence Read Archive under BioProject PRJNA1121973 (accession numbers SAMN41770678-SAMN41770680).

### Statistical analysis

Statistical analyses were performed using PAST4 software [45], with significance set at α = 0.05. Linear regression analyzed correlations between cell concentrations, L33p and L34p gene abundances, and salinity. Differences in TCE degradation rates were assessed using Welch’s t-test. One-way ANOVA followed by Tukey’s post hoc test compared qPCR data, codon usage bias, tRNA gene numbers, and predicted protein pIs across groups. Principal Coordinate Analysis (PCoA) was performed on full-length 16S rRNA gene sequences using QIIME2 (v.2022.2) with Bray–Curtis dissimilarity metrics after rarefaction to 24 810 reads per sample.

## Results

### Effect of salinity on TCE dechlorination in a batch assay

**Figure 1:**
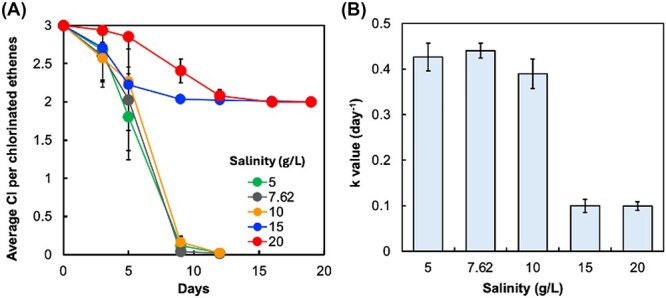
Inhibitory effect of salinity on reductive dechlorination of TCE. (a) TCE dechlorination profiles and (b) first-order dechlorination rate constants (*k*_TCE-to-ETH_) of TCE under different salinity conditions. Experiments were conducted in triplicate. The error bars represent standard deviations (*n* = 3). Salinity stress is expressed as NaCl concentration (g/L).

We first evaluated the effect of salinity on the dechlorination of TCE in a batch assay with TCE-enriched sludge from a saline reactor (7.8 g salt/L). TCE was completely dechlorinated to ethene at a salinity level below 10 g/L within 12 days. At salinity levels of 15 g/L and higher, dechlorination was markedly inhibited and lasted over a 20-day incubation period, with primary accumulation of *cis*-DCE and VC but minor ethene production (Fig. 1(a)). These results indicated different responses of consecutive dechlorination steps to salt stress; the VC-to-ethene step is the most vulnerable. The first-order rate constant *k*_TCE-to-ethene_ remained statistically unchanged (t-test, *P* > .05) at 5–10 g/L salinity but decreased at 15–20 g/L to 0.087 ± 0.01 day^−1^, a reduction of 77.7% (Fig. 1(b)), highlighting the inhibitory effect of high salinity.

### Reactor long-term operation and high salinity

Fed-batch reactor experiments were conducted across a range of salinity levels (Supplementary Table 1). During stage T1 (8.1–8.4 g/L salinity), all reactors achieved complete dechlorination of TCE to ethene via *cis*-DCE and VC intermediates. From stage T2, dechlorination rates increased in the SD reactor as salinity decreased, while in the SI and SM reactors, dechlorination rates remained relatively stable despite increasing salinity levels up to 23.7 g/L (Supplementary Fig. 1, Supplementary Table 2). Maximum dechlorination rates of 103.3–111.9 μmole Cl^−^/L/day were observed at 14.8–23.7 g/L salinity, surpassing those recorded in batch assays. Incremental salinity increases per stage (~4.7 g/L in SI; ~8.6 g/L in SM) did not significantly affect *k*_TCE-to-ethene_ values (t-test, *p* = 0.79). At 31.3 g/L salinity (SM reactor, stage T6), dechlorination persisted but was characterized by a prolonged lag phase, reduced rate, minimal ethene production, and occasional faint-pink coloration of the resazurin redox indicator, indicating insufficient reducing power. Substituting lactate with EOS, which provides a more sustained hydrogen release while maintaining an equivalent electron donor supply, improved *k*_TCE-to-ethene_ from 0.141 to 0.2–0.27 day^−1^. PCoA further revealed that salinity was not the primary driver of microbial community shifts; rather, the substrate change from lactate to EOS had a more pronounced effect on community structure (Supplementary Fig. 2). Despite these adjustments, dechlorination rates decreased by 63.5–68.3% as salinity increased from 23.7 g/L to 31.3–34 g/L.

**Figure 2:**
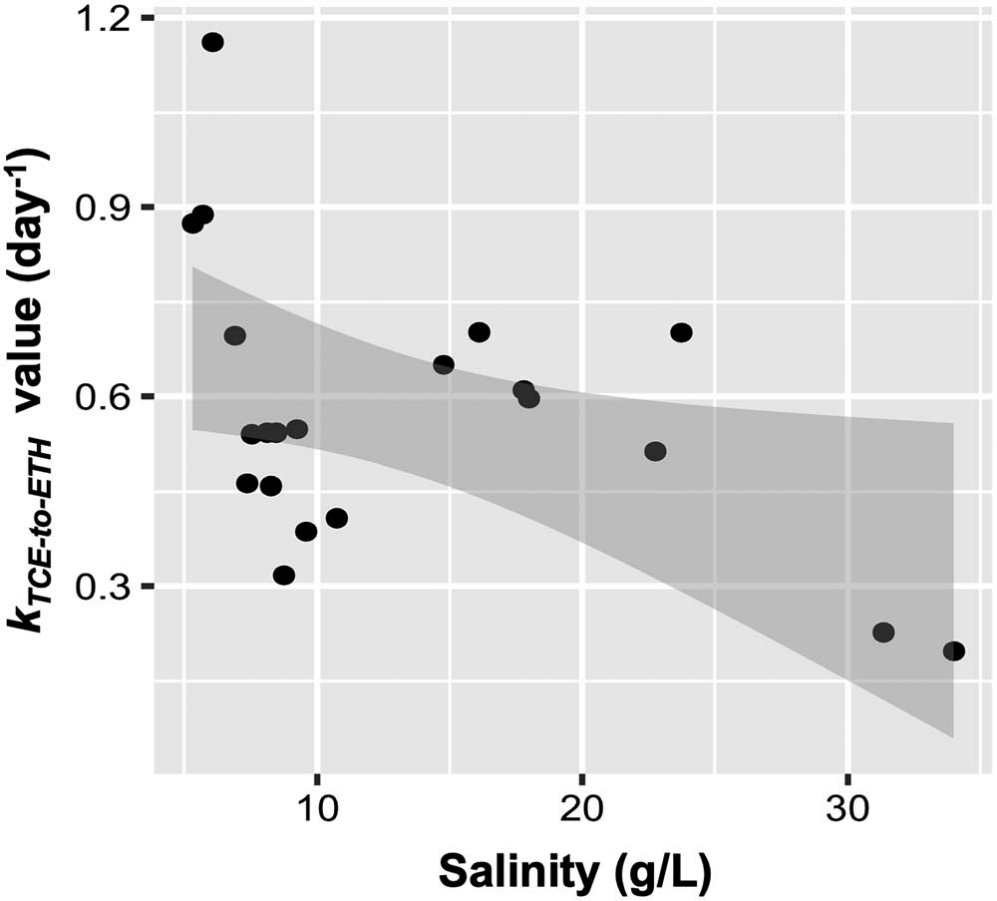
Correlation between salinity and dechlorination rate constant of TCE. Spearman’s correlation analysis revealed a negative correlation (ρ = −0.45, *p* = 0.040) between the first-order dechlorination rate constant (*k*_TCE-to-ETH_) and salinity levels across seven experimental stages (salinity range: 5.3–34 g/L). Each stage is represented by a single data point averaged from the data of multiple batches of TCE dechlorination.

To validate the dechlorination at high salinity, additional batch experiments were conducted using inoculum from the SI reactor. These corroborated complete dechlorination of TCE to ethene at 21 g/L salinity (Supplementary Fig. 3), indicating that prolonged acclimation to elevated salinity substantially improved reductive dechlorination. While overall reactor data showed a modest negative correlation (ρ = −0.45, *p* = 0.04; Fig. 2), rates remained relatively stable or were even enhanced within the moderate salinity range (10–23 g/L). A more pronounced decrease was observed only at higher salinities (>30 g/L), suggesting that dechlorinating microbial communities may have an optimal salinity window, rather than a strictly linear response to increasing salt concentrations.

### High salinity inhibits the activity of *Dehalococcoides* but not that of *Dehalogenimonas*

*Dehalococcoides* and *Dehalogenimonas*, predominant OHRB in the inoculum [30], showed different responses to salinity changes. While their concentrations remained stable in the SD reactor, in SI and SM reactors, they peaked at 16.1–17.8 g/L salinity before decreasing at 34 g/L (Supplementary Fig. 4). *Dehalogenimonas* maintained higher concentrations than *Dehalococcoides* across all salinity levels.

**Figure 3:**
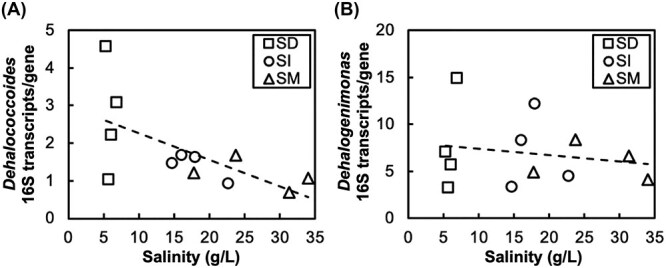
Effects of salinity on the activity of organohalide-respiring bacteria. Correlations were observed between salinity and the specific 16S rRNA gene expression activity of (a) *Dehalococcoides* (ρ = −0.58, p = 0.048) and (b) *Dehalogenimonas* (ρ = −0.03, p = 0.91). Data are representative of stages T4 to T7 in the SD, SM, and SI reactors (salinity range: 6.9–34 g/L). Each point represents one sample per stage per reactor.

*Dehalococcoides* exhibited a strong negative correlation between 16S rRNA transcripts per 16S rRNA gene and salinity (ρ = −0.58, *p* = 0.048) (Fig. 3(a)), indicating reduced transcriptional activity at higher salinity levels. This decrease likely contributes to the observed inhibition of dechlorination at elevated salinity. In contrast, *Dehalogenimonas* maintained relatively stable expression levels across different salinity conditions (ρ = −0.03, *p* = 0.91) (Fig. 3(b)), suggesting greater resistance to salt stress. Notably, our TCE-dechlorinating enrichment contained abundant RDase genes, such as *tceA* and *vcrA* [30]. Regardless of the degree of salinity, the expression level of *tceA* (10^7^ transcripts/ml) was higher than that of *vcrA* (10^5^ transcripts/ml), with both appearing to be unaffected by salinity (one-way ANOVA, *P* > .5, Supplementary Fig. 5). Although high *tceA* and *vcrA* expression activity was observed even at salinity levels of 31–34 g/L, the *k*_TCE-to-ethene_ value considerably decreased (Supplementary Table 2). This suppression of dechlorination activity may not be attributable to the transcriptional process of the *tceA* and *vcrA* genes but rather to their translational process, because the reported RDase enzyme activity of TCE remained unaffected, even at salinity up to 75 g/L [14].

### Phylogeny and genomic characteristics of *Dehalococcoides* and *Dehalogenimonas*

#### Phylogeny

**Figure 4:**
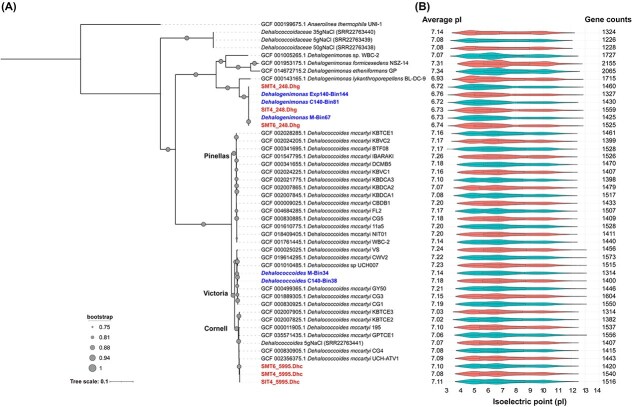
Phylogenomic analysis and protein pI distribution of *Dehalococcoides* and *Dehalogenimonas*. (a) Phylogenomic tree of the MAGs and reference genomes of *Dehalococcoides* and *Dehalogenimonas*. Red bold: Six MAGs identified in this study; blue bold: Five MAGs identified in our previous study; black: 36 relevant complete genomes obtained from NCBI GenBank (accessed in March 2024) and a study on TCE dechlorination [14]. *Anaerolinea thermophila* UNI-1 served as the outgroup. The tree was constructed using GToTree, with 74 bacterial single-copy marker genes obtained from a hidden Markov model and visualized using iTOL (https://itol.embl.de). The scale bar indicates the number of nucleotide substitutions per sequence site. (b) Violin plot of the pI distribution of all predicted proteins in the genomes. Protein pIs were calculated using the EMBOSS pI calculator. The annotated gene counts in each genome are depicted on the right.

Shotgun metagenomic analysis of samples from SI reactor (14.8 g/L salinity) and SM reactor (17.8 and 31.3 g/L salinity) identified six MAGs taxonomically assigned to *Dhc. mccartyi* and *Dehalogenimonas* (Supplementary Table 3). Phylogenomic analysis revealed that the three *Dhc. mccartyi* MAGs clustered closely with the Cornell subgroup (ANI > 98%), contrasting with our previous report of Victoria subgroup predominance in the inoculum [30]. To further investigate this discrepancy, we performed full-length 16S rRNA gene amplicon sequencing on 23 samples using PacBio technology. The results confirmed a compositional shift from the Victoria to Cornell subgroup under high salinity conditions (Supplementary Fig. 6(a)). While *Geobacter* and *Dehalogenimonas* sequences were also detected, only *Dehalogenimonas* emerged as dominant among other OHRB at high salinity (Supplementary Fig. 6(b)). The three *Dehalogenimonas* MAGs clustered together and represented a novel species branching from *Dhg. lykanthroporepellens* BL-DC-9 (ANI: 83.5%, Fig. 4(a)).

#### Protein pI profiles

We explored the pI profiles of proteins predicted in the genomes of *Dehalococcoides* and *Dehalogenimonas* (Fig. 4(b)). The average pI value of the *Dehalogenimonas* MAGs was 6.73 ± 0.01, which is lower than the values of other *Dhg* and *Dhc* genomes. Notably, the average pI value of the *Dehalococcoides* Cornell subgroup was significantly lower (7.07 ± 0.03) than the values of the Pinellas (7.16 ± 0.05) and Victoria (7.19 ± 0.04) subgroups, with *p* values of 3.9 × 10^−5^ and 8.5 × 10^−7^, respectively. According to previous studies, freshwater bacteria exhibit proteins with neutral pIs, while bacteria adapted to high-salt or hypersaline environments tend to encode proteins with low pIs [28, 29]. These pI profiles suggest that *Dehalogenimonas* may have greater ability for salt tolerance compared to *Dehalococcoides*, with the Cornell subgroup potentially having more favorable protein characteristics for salt tolerance among the three subgroups.

### Molecular mechanisms underlying high-salt adaptation

We first examined genes related to osmolyte synthesis and transport, sodium efflux, and potassium uptake in *Dehalococcoides* and *Dehalogenimonas* genomes (Supplementary Fig. 7). The Cornell subgroup lacked *mscL* (potassium uptake) and *ocd* (proline synthesis) genes, suggesting lower osmoadaptation potential compared to Pinellas and Victoria subgroups. *Dehalogenimonas* exhibited higher diversity in osmolyte transport, ectoine synthesis, and sodium efflux genes than *Dehalococcoides*. This osmoadaptation gene feature aligns with experimental results, suggesting that *Dehalogenimonas* exhibits greater adaptability to high-salt environments. However, these findings do not support that the Cornell subgroup had stronger survival abilities than the Victoria subgroup did at high degrees of salinity (Supplementary Fig. 4). Therefore, the high degree of tolerance to salinity observed in the Cornell subgroup may be attributable to factors beyond the mere existence of osmoadaptation genes in their genomes.

#### Consistency in tRNA and anticodon profiles

To further explore the molecular mechanisms potentially underlying high-salt adaptation, as indicated by our experimental results, we examined the genes involved in the translation processes. Regarding the 1780 predicted tRNA loci identified in the analyzed genomes, no significant variations in the number of tRNA genes were observed between *Dehalococcoides* and *Dehalogenimonas*. (Supplementary Tables 4, 5, 6, and  7). We identified 57 anticodons linked to the standard amino acids, with these anticodons present in more than 90% of the genomes (Supplementary Fig. 8). This conservation of tRNA gene repertoire and anticodon diversity indicated a fundamental similarity in the core translation machinery among these species, leading us to investigate additional mechanisms that might explain their varying salt tolerance.

#### *Dehalococcoides* exhibit codon usage bias at the subspecies level

Our subsequent analysis uncovered their variations in codon usage patterns at the subspecies level within *Dehalococcoides*. We first examined the average codon adaptation index (CAI_ave_) values across all genes in every *Dehalococcoides* genome. These values exhibited uniform distributions across all genomes of *Dehalococcoides*, with a CAI_ave_ of 0.18 ± 0.001 (Supplementary Fig. 9), which is considerably lower than the value (0.59 ± 0.097) reported for 1169 prokaryotic genomes in a previous study [46]. These findings suggest that *Dehalococcoides* strains with low CAI_ave_ values prefer specific codons in highly expressed genes, with other genes utilizing a broader range of codons. At the genomic level, *Dehalococcoides* strains exhibit asymmetry and preference in codon usage, which may be associated with optimal protein synthesis efficiency and resource allocation. To further examine codon usage preferences, we analyzed the codon frequency of all *Dehalococcoides* strains for the 22 amino acids, categorizing them into three subgroups. Compared with the Pinellas and Victoria subgroups, the Cornell subgroup exhibited significantly higher usage of 22 preferred codons for 15 amino acids (*p* < 0.05, Supplementary Fig. 10). By contrast, the Pinellas and Victoria subgroups exhibited significantly higher usage of 23 and 21 preferred codons, respectively, for 15 amino acids compared with the Cornell subgroup (*p* < 0.05). These findings highlight distinct variations in codon usage bias across different subgroups, reflecting divergent genomic features among *Dehalococcoides* subspecies.

#### Distribution of ribosomal genes

Genes encoding three rRNA (Supplementary Fig. 11), 20 small-subunit (SSU, Supplementary Fig. 12(a)), and 33 large-subunit (LSU, Supplementary Fig. 12(b)) ribosomal proteins were identified in the genomes of *Dehalococcoides* and *Dehalogenimonas*, with nearly all ribosomal protein genes existing as single copies. Notably, the gene encoding the zinc-dependent SSU ribosomal protein S14P (S29e) was only detected in the majority of the *Dehalogenimonas* genomes (Supplementary Fig. 12(a)). The LSU ribosomal protein L34p gene was absent in several strains from the Cornell and Victoria subgroups. A prevalence of the zinc-dependent L33p gene was observed only in the Cornel subgroup and *Dehalogenimonas*, but the Victoria and Pinellas subgroups lack this gene (Supplementary Fig. 12(b)).

**Figure 5:**
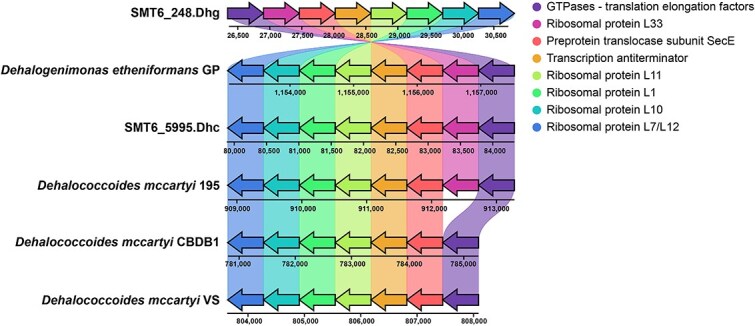
Comparative genomic analysis of the L33p gene operon structure in *Dehalococcoides* and *Dehalogenimonas* strains. Synteny map of the L33p gene operon and surrounding genomic regions in representative strains of *Dehalococcoides* and *Dehalogenimonas*. The analysis was performed using OperonMapper for operon prediction and visualized with Clinker & Clustermap.Js. Colored arrows represent different genes: Purple for GTPases-translation elongation factors, pink for ribosomal protein L33, red for preprotein translocase subunit SecE, orange for transcription antiterminator, and various shades of green and blue for other ribosomal proteins (L11, L1, L10, L7/L12). Genomic positions are shown in base pairs below each strain. Strains analyzed include SMT6_248.Dhg (MAG), *Dehalococcoides mccartyi* 195 (Cornell subgroup), *Dhc. mccartyi* CBDB1 (Pinellas subgroup), and *Dhc. mccartyi* VS (Victoria subgroup), *Dehalogenimonas etheniformans* GP, and SMT6_5995.Dhc (MAG). The figure demonstrates the highly conserved operon structure across these genera, with the notable absence of the L33p gene in the Victoria (VS) and Pinellas (CBDB1) subgroups of *Dehalococcoides*.

Comparative genomic analysis reveals a highly conserved operon structure across *Dehalococcoides* and *Dehalogenimonas* genomes, harboring genes encoding four LSU ribosome proteins (L1, L7/L12, L10, and L11), translation elongation factor, preprotein translocase subunit SecE, and a transcription antiterminator (Fig. 5). The lack of the L33p gene in the Victoria (represented by strain VS) and Pinellas (represented by strain CBDB1) subgroups, while maintaining the exact operon structure, underscores the specificity of this gene loss and suggests an evolutionary significance in the genomes of *Dehalococcoides* lineages. *Dehalogenimonas* and the Cornell subgroup shared 73.68% amino acid sequence similarity in L33p, indicating a common origin and functional conservation. Intriguingly, the abundance of the L33p gene in our reactor metagenomes corresponded to increasing salt levels, suggesting its association with salt tolerance. Although the L34p gene also exhibited a positive correlation with salinity, this relationship was notably weaker (β = 3 × 10^−5^, R^2^ = 0.35) compared to L33p (β = 2 × 10^−4^, R^2^ = 0.78), further supporting the hypothesis that L33p may play a more specialized role in salt adaptation (Supplementary Fig. 13(a)). At high salinity (31.3 g/L), L33p-containing MAGs were distributed across diverse bacterial phyla, including Proteobacteria (22 MAGs), Actinobacteriota (10 MAGs), Bacteroidota (8 MAGs), and Spirochaetota (8 MAGs), among others, representing a substantial portion of the 74 medium- to high-quality MAGs (>50% completeness and < 10% contamination) recovered from the SM reactor (Supplementary Fig. 13(b)).

Further investigation through NCBI blastp search revealed a broad distribution of L33p, with 140 homologous protein sequences predominantly within phylum Chloroflexota, including Dehalococcoidia (64 sequences), Chloroflexota (33 sequences), Anaerolineales (17 sequences), and Anaerolineae (11 sequences), along with representatives from other class-level taxa (15 sequences). The L33p gene was also identified in *Wenzhouxiangella* sp. and within Pseudomonadota and Acidobacteriota. STRING database analysis showed that while L33p is not universally prevalent among microorganisms, it exhibits significant co-occurrence with several specific ribosomal proteins (L11, L1, L10, L7/L12, L13, S18, L28, L21, and S10) (Supplementary Fig. 14), suggesting a coordinated evolutionary trajectory. This widespread distribution across diverse bacterial lineages in salinity-gradient environments indicates that L33p-mediated salt adaptation may be a general feature among halotolerant bacteria.

## Discussion

Our study demonstrates distinct responses to salinity in TCE dechlorination, highlighting the remarkable adaptability of *Dehalococcoides* Cornell subgroup and *Dehalogenimonas.* While previous studies using pure cultures (e.g. *Dhg. etheniformans* GP [16], *Dhc. mccartyi* NIT01 [13], CG and MB [14]), and batch assays in this study reported limited dechlorination at salinities above 15 g/L, our reactor consortia, following extended acclimation, achieved complete dechlorination to ethene at salinities as high as 31.3 g/L using EOS. This enhanced performance is likely attributed not only to salt-tolerant OHRB but also to the effectiveness of the gradual acclimation strategy and the resulting microbial community structure that sustained OHRB activity. Our findings also extend previous work on halophilic *Dehalobium chlorocoercia* DF-1, originally isolated from estuarine sediments, which thrives in marine salinity and dechlorinates polychlorinated biphenyl [47], yet shows limited chloroethene dechlorination, restricted to PCE-to-DCE conversion without ethene production [48]. Overall, this work advances the understanding of complete TCE reductive dechlorination under brackish to marine conditions, providing new insights into the ecological potential and limits of high-salinity dechlorinating consortia.

As indicated by our experimental findings, the reduced dechlorination activity observed in *Dehalococcoides* at high salt concentrations is likely due to translation-related limitations rather than transcriptional inhibition. While the transcription of *tceA* and *vcrA* genes remained at high levels under high-salinity conditions, and RDase enzymes can retain their activity in high-salt environments [14], the overall dechlorination performance decreased. This suggests potential protein synthesis or stability disruptions, aligning with known microbial responses to environmental stress [49]. High salinity can trigger riboswitch folding and alarmone (ppGpp) synthesis during translation, influencing cell physiology [50]. Additionally, protein misfolding and aggregation at high salinity [51] and altered functionality of chemical chaperones (e.g., glycine betaine, glycerol, and proline) can reduce protein stability and increase enzyme inactivation [52, 53]. The findings of this study suggest that salinity stress may primarily affect the translation process or protein stability of RDase enzymes rather than their intrinsic activity. Further research is required to explore the specific effects of high salinity on RDase protein synthesis and stability.

Overall, our experimental and genomic analysis results revealed variations in the degree of tolerance to salinity between *Dehalococcoides* and *Dehalogenimonas*. Specifically, *Dehalogenimonas* spp. have more comprehensive genetic mechanisms for osmolyte accumulation and salt-in osmotic balance, which enhance their osmotic regulation and salt tolerance (Supplementary Fig. 7). These mechanisms aid in maintaining osmotic homeostasis, regulating essential ion concentrations, and mitigating ion toxicity, thereby ensuring cellular viability and metabolic stability in high-salt environments. These findings help explain the high abundance of *Dehalogenimonas* in reactors with high salinity, and align with a recent report showing its growth at salinities up to 50 g/L [17]. This suggests that *Dehalogenimonas* may play a more prominent role in chlorinated solvent degradation in saline environments. By contrast, *Dehalococcoides* spp. have few genes related to salt tolerance (Supplementary Fig. 7), which complicates the process of alleviating the adverse effects of high salinity on the intracellular environment. This genomic deficiency explains why strains from the Victoria and Pinellas subgroups exhibit poor tolerance to salt stress in pure culture tests [13, 14] and why *Dehalococcoides* is more commonly reported in freshwater groundwater environments [8]. However, it does not explain the high dechlorination efficiency and growth of the Cornell subgroup at a salinity level of 23–31 g/L observed in this study. The Cornell subgroup strain MB maintains substantial cell density under high-salt conditions [14], supporting its salt tolerance potential despite reduced dechlorination activity. Notably, we observed a shift in the population of *Dehalococcoides* from the Victoria to the Cornell strain in the long-term operated inoculum reactor [30]. As the salinity levels in the inoculum reactor gradually increased over more than 2 years in fed-batch mode, the dominance of Victoria strains was progressively replaced by Cornell strains. This different salt tolerance likely reflects their evolutionary history and adaptation to distinct environmental niches. Together, these results showed the superior salt tolerance of the Cornell subgroup and suggested that prolonged exposure to increasing salinity can selectively enrich more salt-tolerant *Dehalococcoides* strains within microbial communities.

Our study reveals intriguing differences in codon usage preferences among *Dehalococcoides* subgroups. While the genetic code is largely conserved across life, the frequency of synonymous codon usage can vary significantly between microorganisms, often correlating with genomic GC content bias [54]. The Cornell subgroup showed significantly different GC content compared to the Victoria and Pinellas subgroups. It also showed markedly lower average protein pI values (Fig. 4(b)), while reduced pI values have been linked to halophilic adaptation in other microorganisms [28, 41, 55]. The Cornell subgroup exhibits a distinct pattern characterized by an increased prevalence of rare codons encoding aspartate, arginine, glycine, glutamine, leucine, proline, serine, and valine (Supplementary Fig. 10). Notably, this unique codon usage profile partially aligns with patterns observed in known halophilic prokaryotes [56], particularly in the preferential use of CGG for arginine and GUC for valine. These similarities in codon usage suggest a potential link to the Cornell subgroup’s superior salt tolerance compared to the Victoria and Pinellas subgroups, indicating its convergent evolution in codon optimization strategies for salt adaptation. This genomic characteristic resonates with Di Giulio’s (2005) findings on the relationship between amino acid usage and adaptation to high-pressure environments [57]. Such environment-specific codon optimization has been observed in other extremophiles, such as the psychrophilic bacterium *Psychromonas ingrahamii*, which employs distinct codon preferences for efficient translation at low temperatures [58]. The novel finding reveals significant differences in genomic evolution even among closely related *Dehalococcoides* strains, highlighting their remarkable evolutionary capacity to respond to specific environmental pressures. While our results provide multifaceted evidence for salt adaptation at the codon usage level, further experimental validation is crucial to establish direct causal links between these codon preferences and specific cellular functions under varying salt concentrations.

Our data reveals a striking correlation between L33p gene abundance and increasing salinity levels (Supplementary Fig. 13(a)), aligning with a recent study reporting elevated L33p expression in yeast under salt stress [59]. Given its zinc-finger motif, this small ribosomal protein may be associated with the structural stability of the ribosome, potentially enhancing translational efficiency under osmotic stress [58, 60]. Our analysis showed that L33p is present in the Cornell subgroup but absent in Victoria and Pinellas subgroups, despite conservation of the surrounding operon structure, suggesting a subgroup-specific gene loss likely driven by selective evolutionary pressures. The absence of L33p in the latter groups may reflect genome streamlining, a phenomenon also observed in other bacterial lineages that have lost specific ribosomal protein genes [61, 62]. Considering the compact genome of *Dehalococcoides* (~1.4 Mbp), the Victoria and Pinellas subgroups may have discarded non-essential genes for survival in more stable, terrestrial environments [8]. In contrast, the retention of L33p in the Cornell subgroup may support its adaptation to fluctuating or saline conditions. Notably, L33p is also distributed across a wide range of bacterial phyla from saline environments (Supplementary Fig. 14), suggesting a broader role in salt adaptation. While these observations provide compelling correlative evidence, experimental validation through targeted gene manipulation will be necessary to confirm the functional role of L33p in salt tolerance.

In this study, we experimentally and genomically elucidated the evolutionary basis of salt tolerance differences between *Dehalococcoides* subgroups and *Dehalogenimonas*. The Cornell subgroup of *Dehalococcoides* and *Dehalogenimonas* have evolved salt tolerance mechanisms through the synergy of osmoadaptation-related genes and distinctive protein synthesis strategies. These likely support effective TCE dechlorination in high-salinity environments, providing a mechanistic foundation for developing salt-tolerant and highly effective dechlorination agents. The observed correlation between gene translation and salt tolerance in OHRB at the (sub) species level advances our understanding of organohalide respiration under saline conditions and sheds light on prokaryotic stress adaptation. Collectively, these findings deepen the understanding of microbial evolution and offer a valuable framework for developing more resilient bioremediation technologies to address global environmental challenges.

## Supplementary Material

SI_ISMEJ_comm_rev_ycaf101

## Data Availability

All data are available in the main text or the supplementary materials. The full-length 16S rRNA gene amplicon sequencing and shotgun metagenomic datasets generated in this study have been deposited in the Sequence Read Archive of the National Center for Biotechnology Information under BioProject ID PRJNA1121973.
