# Supplementary material for: Genomic personalities of Dehalococcoides subspecies and Dehalogenimonas illuminate complete trichloroethene dechlorination in high-salt conditions
Source: ISME Commun. 2025 Jun 19;5(1):ycaf101. doi: 10.1093/ismeco/ycaf101 (PMC12415852; doi:10.1093/ismeco/ycaf101)
Supplement: SI_ISMEJ_comm_rev_ycaf101 [file si_ismej_comm_rev_ycaf101.pdf]

Genomic Personalities of *Dehalococcoides* Subspecies and *Dehalogenimonas* Illuminate Complete Trichloroethene Dechlorination in High-Salt Conditions

Wei-Yu Chen[1], Yun-Chi Lan[1], Jiung-Wen Chen[2], Jer-Horng Wu[1*]

[1]Department of Environmental Engineering, National Cheng Kung University, Taiwan

[2]Department of Biology, University of Alabama at Birmingham, Birmingham, AL, USA

*Corresponding author

Tel: (886)910385159

Fax: (886)62752790

E-mail: enewujh@ncku.edu.tw

Postal address: No. 1, University Road, East District, Tainan City 701, Taiwan

DESCRIPTION OF SUPPORTING INFORMATION

Supplementary Table 1 Operating parameters for the bench-scale sequencing batch reactors
Supplementary Table 2 Kinetics of TCE dechlorination at various salinities in the reactors.
Supplementary Table 3 *Dehalococcoides* and *Dehalogenimonas* MAGs from reactors at different salinity levels.
Supplementary Table 4 Genome statistics of Cornell subgroup of *Dehalococcoides mccartyi*
Supplementary Table 5 Genome statistics of Victoria subgroup of *Dehalococcoides mccartyi*
Supplementary Table 6 Genome statistics of Pinellas subgroup of *Dehalococcoides mccartyi*
Supplementary Table 7 Genome statistics of *Dehalogenimonas*
Supplementary Fig. 1 TCE dechlorination in the (a) SD, (b) SI, and (c) SM reactors.
Supplementary Fig. 2 Principal coordinate analysis (PCoA) of microbial communities in the three reactors.
Supplementary Fig. 3 Complete TCE dechlorination at a salinity level of 21 g/L.
Supplementary Fig. 4 Effects of salinity on *Dehalococcoides* and *Dehalogenimonas* 16S rRNA gene abundance
Supplementary Fig. 5 Expression activities of *vcrA* and *tceA* genes at different degrees of salinity.
Supplementary Fig. 6 Phylogenetic tree and community dynamics of organohalide-respiring bacteria in reactors.
Supplementary Fig. 7 Distribution and number of copies of salt tolerance–related genes in the genomes.
Supplementary Fig. 8 Distribution and copy number of tRNA genes in the genomes.
Supplementary Fig. 9 Distribution of codon adaptation index (CAI_{ave}) values across *Dehalococcoides* strains.
Supplementary Fig. 10 Distinct codon usage preferences in different subgroups of *Dehalococcoides*.
Supplementary Fig. 11 Schematic representation of rRNA gene locations.
Supplementary Fig. 12 Distribution and copy number of ribosomal protein genes in the genomes.
Supplementary Fig. 13 Correlation between salinity and LSU ribosomal protein gene abundance, and taxonomic distribution of L33p-containing MAGs.
Supplementary Fig. 14 Gene co-occurrence analysis revealing the distribution pattern of ribosomal protein L33p.

Supplementary Table 1 Operating parameters for the bench-scale sequencing batch reactors

SD Reactor

Stage	T1	T2	T3	T4	T5	T6	T7
Operation period (day)[a]	29	40	31	22	17	26	22
Salinity (g /L)	8.4	7.5	7.4	6.9	6.1	5.7	5.3
TCE concentration (mM) per feeding cycle[b] × cycle	0.152×2<br>0.228×1	0.228×4	0.228×3	0.228×3	0.228×3	0.228×4	0.228× 3
Substrate	Lactate	Lactate	Lactate	Lactate	Lactate	Lactate	Lactate
pH	7.17	7.15	7.12	7.05	7.01	7.06	7.08
Temperature (°C)	29.1	29.7	29.7	29.9	29.9	30.1	29.4
Cell retention time (Day)	261	360	279	198	153	234	198

SI Reactor

Stage	T1	T2	T3	T4	T5	T6	T7
Operation period (day)	29	40	31	22	17	26	22
Salinity (g /L)	8.1	9.6	10.7	14.8	16.1	18.0	22.7
TCE concentration (mM) per feeding cycle × cycle	0.152×2<br>0.228×1	0.228×3<br>0.114×2	0.228×3	0.228×3	0.228×3	0.228×4	0.228× 3
Substrate	Lactate	Lactate	Lactate	Lactate	Lactate	Lactate	Lactate
pH	7.15	7.14	7.11	7.06	7.02	7.04	7.05
Temperature (°C)	29.6	30.2	30.1	29.9	30	29.9	29.5
Cell retention time (Day)	261	360	279	198	153	234	198

SM Reactor

Stage	T1	T2	T3	T4	T5	T6	T7
Operation period (day)	29	40	31	22	17	38	49
Salinity (g /L)	8.3	8.7	9.2	17.8	23.7	31.3	34.0
TCE concentration (mM) per feeding cycle × cycle	0.152×2<br>0.228×1	0.457×1<br>0.228×2	0.228×3	0.228×3	0.228×3	0.228×4	0.228× 3
Substrate	Lactate	Lactate	Lactate	Lactate	Lactate	Lactate[c] & EOS[d]	EOS
pH	7.17	7.22	7.18	7.10	7.08	7.07	7.00
Temperature (°C)	29.3	29.8	29.7	29.8	29.9	29.7	29.5
Cell retention time (Day)	261	360	279	198	153	342	441

[a]Each salinity stage included 3-5 feeding cycles, with stage duration shown in 'Operation period'
[b]Each feeding cycle represents a complete process of TCE addition followed by dechlorination.
[c]The first cycle of lactate added to the SM reactor serves as an electron donor.
[d]The second and third cycles of emulsified oil added to the SM reactor serves as electron donors.

Supplementary Table 2 Kinetics of TCE dechlorination at various salinities in the reactors.

Reactor	Stage	Salinity (g/L)	Substrate	Dechlorination rate (µmole Cl^- /L/day)	$k_{TCE\text{-}to\text{-}ethene}$ (day^{-1})	R^2
SD	T1	8.4	Lactate	74.3 ± 33.3	0.54 ± 0.16	0.96
	T2	7.5	Lactate	97.8 ± 0.0	0.54 ± 0.02	0.93
	T3	7.4	Lactate	76.2 ± 15.5	0.46 ± 0.22	0.91
	T4	6.9	Lactate	121.8 ± 10.8	0.70 ± 0.11	0.96
	T5	6.1	Lactate	184.8 ± 61.5	1.16 ± 0.36	0.97
	T6	5.7	Lactate	117.4 ± 19.6	0.90 ± 0.18	0.91
	T7	5.3	Lactate	127.7 ± 31.5	0.88 ± 0.25	0.94
SI	T1	8.1	Lactate	74.3 ± 33.3	0.54 ± 0.14	0.96
	T2	9.6	Lactate	97.8 ± 0.0	0.39 ± 0.04	0.95
	T3	10.7	Lactate	76.2 ± 15.5	0.41 ± 0.09	0.92
	T4	14.8	Lactate	104.6 ± 13.5	0.65 ± 0.10	0.97
	T5	16.1	Lactate	111.9± 18.5	0.72 ± 0.13	0.96
	T6	18.0	Lactate	107.6 ± 16.9	0.60 ± 0.28	0.77
	T7	22.7	Lactate	103.3 ± 7.7	0.51 ± 0.04	0.83
SM	T1	8.3	Lactate	74.3 ± 33.3	0.46 ± 0.09	0.95
	T2	8.7	Lactate	117.4 ± 19.6	0.32 ± 0.02	0.98
	T3	9.2	Lactate	78.7 ± 14.6	0.55 ± 0.12	0.92
	T4	17.8	Lactate	104.6 ± 13.5	0.61 ± 0.14	0.89
	T5	23.7	Lactate	111.9± 18.5	0.63 ± 0.08	0.88
	$T6_{Lac}$[a]	31.3	Lactate	68	0.141	0.90
	$T6_{EOS}$[b]	31.3	Emulsified oil	56	0.27	0.88
	T7	34.0	Emulsified oil	51.6 ± 3.8	0.20 ± 0.03	0.73

[a] Lactate was added to the SM reactor as an electron donor in the first batch of stage T6.
[b] Emulsified oil was added to the SM reactor as electron donors in the second and third batches of stage T6.

Supplementary Table 3 Characterization of *Dehalococcoides* and *Dehalogenimonas* MAGs recovered from reactors at different salinity levels: SI reactor stage T4 (14.8 g/L), SM reactor stage T4 (17.8 g/L), and SM reactor stage T6 (31.3 g/L).

MAG	Taxonomy	Completeness (%)	Contamination (%)	Contig	Genome size (Mbp)	Contig N50 (bp)	CDS
SIT4_5995.Dhc	*Dehalococcoides*	99.01	0	4	1.4	562636	1516
SMT4_5995.Dhc	*Dehalococcoides*	99.01	0	4	1.5	550995	1541
SMT6_5995.Dhc	*Dehalococcoides*	99.01	0	5	1.4	520525	1420
SIT4_248.Dhg	*Dehalogenimonas*	85.81	1.98	42	1.6	57788	1559
SMT4_248.Dhg	*Dehalogenimonas*	80.86	0	38	1.5	67469	1460
SMT6_248.Dhg	*Dehalogenimonas*	86.8	0	41	1.5	56032	1525

Supplementary Table 4 Genome statistics of Cornell subgroup of *Dehalococcoides mccartyi*

**Cornell Subgroup**	SIT4_5995.Dhc	SMT4_5995.Dhc	SMT6_5995.Dhc	UCH-ATV1	KBTCE3	KBTCE2	CG4	195	GPTCE1	Dhc_5gNaCl
Accession	PRJNA1121973	PRJNA1121973	PRJNA1121973	NZ_AP017649	NZ_CP019866	NZ_CP019865	NZ_CP006950	NC_002936	NZ_CP141531	SRR22763441
Genome size (bp)	1436856	1457659	1365910	1387782	1271604	1329198	1382308	1469720	1494874	1364399
Contig	4	4	5	1	1	1	1	1	1	4
DNA G+C (%)	48.8	48.8	48.9	48.8	49.3	49.1	48.7	48.9	48.5	48.9
tRNA G+C (%)	60.6	59.5	58.9	60.55	60.6	60.3	60.46	59.6	60.5	60.5
Contig N50 (bp)	562636	550995	520525	1387782	1271604	1329198	1382308	1469720	1494874	694932
Coding density (%)	90.9	91.1	90.8	91.3	91.9	91.8	90.8	91.1	91.1	91.2
tRNA	49	51	52	47	45	46	47	49	47	46
tmRNA	1	1	1	1	1	1	1	1	1	1
rRNA	3	5	4	3	3	3	3	3	3	4
ncRNA	1	1	1	1	1	1	1	1	1	1
ncRNA regions	9	9	9	9	8	8	8	9	9	9
CRISPR arrays	0	0	0	0	0	0	0	0	0	0
Protein-coding genes	1516	1541	1420	1443	1314	1382	1415	1537	1569	1407
Pseudogenes	1	2	1	0	1	1	1	1	4	2
Hypotheticals	91	137	69	71	41	45	42	27	116	52
Signal peptides	0	0	0	0	0	0	0	0	0	0
sORFs	0	0	0	0	0	0	0	0	0	0
Gaps	0	0	0	0	0	0	0	0	0	0
oriCs	1	1	1	1	1	1	1	1	1	1
oriVs	0	0	0	0	0	0	0	0	0	0
oriTs	0	0	0	0	0	0	0	0	0	0

Supplementary Table 5 Genome statistics of Victoria subgroup of *Dehalococcoides mccartyi*

**Victoria Subgroup**	C140-Bin38	UCH007	CWV2	CG3	CG1	GY50	VS
Accession	ERA20758382	NZ_AP014722	NZ_CP080651	NZ_CP013074	NZ_CP006949	NC_022964	NC_013552
Genome size (bp)	1351640	1473548	1439600	1521287	1486678	1407418	1413462
Contig	3	1	1	1	1	1	1
DNA G+C (%)	47.1	46.9	47.1	46.9	46.9	47.2	47.3
tRNA G+C (%)	60.3	60.5	60.3	60.7	59.9	58.8	60.5
Contig N50 (bp)	902609	1473548	1439600	1521287	1486678	1407418	1413462
Coding density (%)	91.1	91.1	91.1	90.7	90.3	90.8	91.2
tRNA	48	50	48	50	51	47	47
tmRNA	1	1	1	1	1	1	1
rRNA	3	3	3	3	3	3	3
ncRNA	3	2	2	1	1	1	1
ncRNA regions	9	9	9	9	9	9	9
CRISPR arrays	0	2	1	1	0	0	0
Protein-coding genes	1398	1515	1573	1604	1550	1446	1455
Pseudogenes	4	1	80	2	1	2	2
Hypotheticals	30	13	90	41	20	43	11
Signal peptides	0	0	0	0	0	0	0
sORFs	0	0	0	0	0	0	0
Gaps	0	0	0	0	0	0	0
oriCs	1	1	1	1	1	1	1
oriVs	0	0	0	0	0	0	0
oriTs	0	0	0	0	0	0	0

Supplementary Table 6 Genome statistics of Pinellas subgroup of *Dehalococcoides mccartyi*

**Pinellas Subgroup**	IBARAKI	DCMB5	KBVC1	KBDCA3	KBDCA2	KBDCA1	BTF08	KBTCE1	KBVC2
Accession	NZ_AP014563	NC_020386	NZ_CP019968	NZ_CP019946	NZ_CP019868	NZ_CP019867	NC_020387	NZ_CP019999	NZ_CP019969
Genome size (bp)	1451056	1431902	1359904	1337486	1394319	1428463	1452335	1388914	1337731
Contig	1	1	1	1	1	1	1	1	1
DNA G+C (%)	47.0	47.1	47.3	47.6	47.5	47.4	47.3	47.3	47.2
tRNA G+C (%)	60.5	60.5	60.5	59.8	59.6	59.4	59.8	59.9	59.9
Contig N50 (bp)	1451056	1431902	1359904	1337486	1394319	1428463	1452335	1388914	1337731
Coding density (%)	90.7	90.7	90.5	91.6	91.0	91.0	91.1	91.1	90.9
tRNA	49	47	47	47	49	50	49	50	50
tmRNA	1	1	1	1	1	1	1	1	1
rRNA	3	3	3	3	3	3	3	3	3
ncRNA	1	1	1	1	1	1	1	1	1
ncRNA regions	7	7	8	7	7	7	7	7	7
CRISPR arrays	0	2	1	1	0	0	0	0	0
Protein-coding genes	1526	1470	1407	1398	1479	1517	1528	1461	1399
Pseudogenes	11	2	0	3	4	4	2	2	2
Hypotheticals	57	20	11	32	58	58	34	43	39
Signal peptides	0	0	0	0	0	0	0	0	0
sORFs	0	0	0	0	0	0	0	0	0
Gaps	0	0	0	0	0	0	0	0	0
oriCs	1	1	1	1	1	1	1	1	1
oriVs	0	0	0	0	0	0	0	0	0
oriTs	0	0	0	0	0	0	0	0	0

Supplementary Table 6 (*continued*)

**Pinellas Subgroup**	CBDB1	NIT01	WBC-2	11a5	CG5	FL2
Accession	NC_007356	NZ_AP024514	NZ_CP017572	NZ_CP011127	NZ_CP006951	NZ_CP038470
Genome size (bp)	1395502	1342191	1374583	1461973	1362151	1422358
Contig	1	1	1	1	1	1
DNA G+C (%)	47.0	47.3	47.4	46.9	47.2	47.0
tRNA G+C (%)	59.0	60.5	60.4	60.6	60.6	60.4
Contig N50 (bp)	1395502	1342191	1374583	1461973	1362151	1422358
Coding density (%)	90.6	90.8	91.1	90.3	90.6	90.5
tRNA	47	46	48	50	47	48
tmRNA	1	1	1	1	1	1
rRNA	3	3	3	3	3	3
ncRNA	1	1	1	1	1	1
ncRNA regions	8	8	7	8	8	8
CRISPR arrays	1	0	0	1	0	0
Protein-coding genes	1433	1411	1440	1528	1409	1507
Pseudogenes	1	5	0	0	0	3
Hypotheticals	30	29	68	6	13	38
Signal peptides	0	0	0	0	0	0
sORFs	0	0	0	0	0	0
Gaps	0	2	0	0	0	0
oriCs	1	1	1	1	1	1
oriVs	0	0	0	0	0	0
oriTs	0	0	0	0	0	0

Supplementary Table 7 Genome statistics of *Dehalogenimonas*

	SIT4_248.Dhg	SMT4_248.Dhg	SMT6_248.Dhg	*lykanthroporepellens* BL-DC-9	*etheniformans* GP	*formicexedens* NSZ-14	WBC-2
Accession	PRJNA1121973	PRJNA1121973	PRJNA1121973	NC_014314	NZ_CP110635	NZ_CP018258	NZ_CP011392
Genome size (bp)	1570235	1459741	1527835	1686510	2068322	2092789	1725728
Contig	42	38	41	1	1	1	1
DNA G+C (%)	56.5	56.1	56.0	55.0	51.9	54.0	49.2
tRNA G+C (%)	60.5	60.7	60.6	60.9	61.0	60.5	60.5
Contig N50 (bp)	5778	67469	56032	1686510	2068322	2092789	1725728
Coding density (%)	89.7	90.0	89.9	89.7	88.2	89.2	88.4
tRNA	46	45	45	47	48	51	48
tmRNA	1	0	1	1	1	1	1
rRNA	4	1	3	3	3	3	3
ncRNA	2	1	1	1	1	1	1
ncRNA regions	13	13	13	10	13	1	9
CRISPR arrays	0	0	0	0	0	0	0
Protein-coding genes	1559	1460	1525	1715	2065	2155	1727
Pseudogenes	0	0	0	1	1	3	0
Hypotheticals	342	342	343	406	102	29	113
Signal peptides	0	0	0	0	0	0	0
sORFs	0	0	0	0	0	0	0
Gaps	0	0	0	0	0	0	0
oriCs	0	0	0	0	0	0	0
oriVs	0	0	0	0	0	0	0
oriTs	0	0	0	0	0	0	0

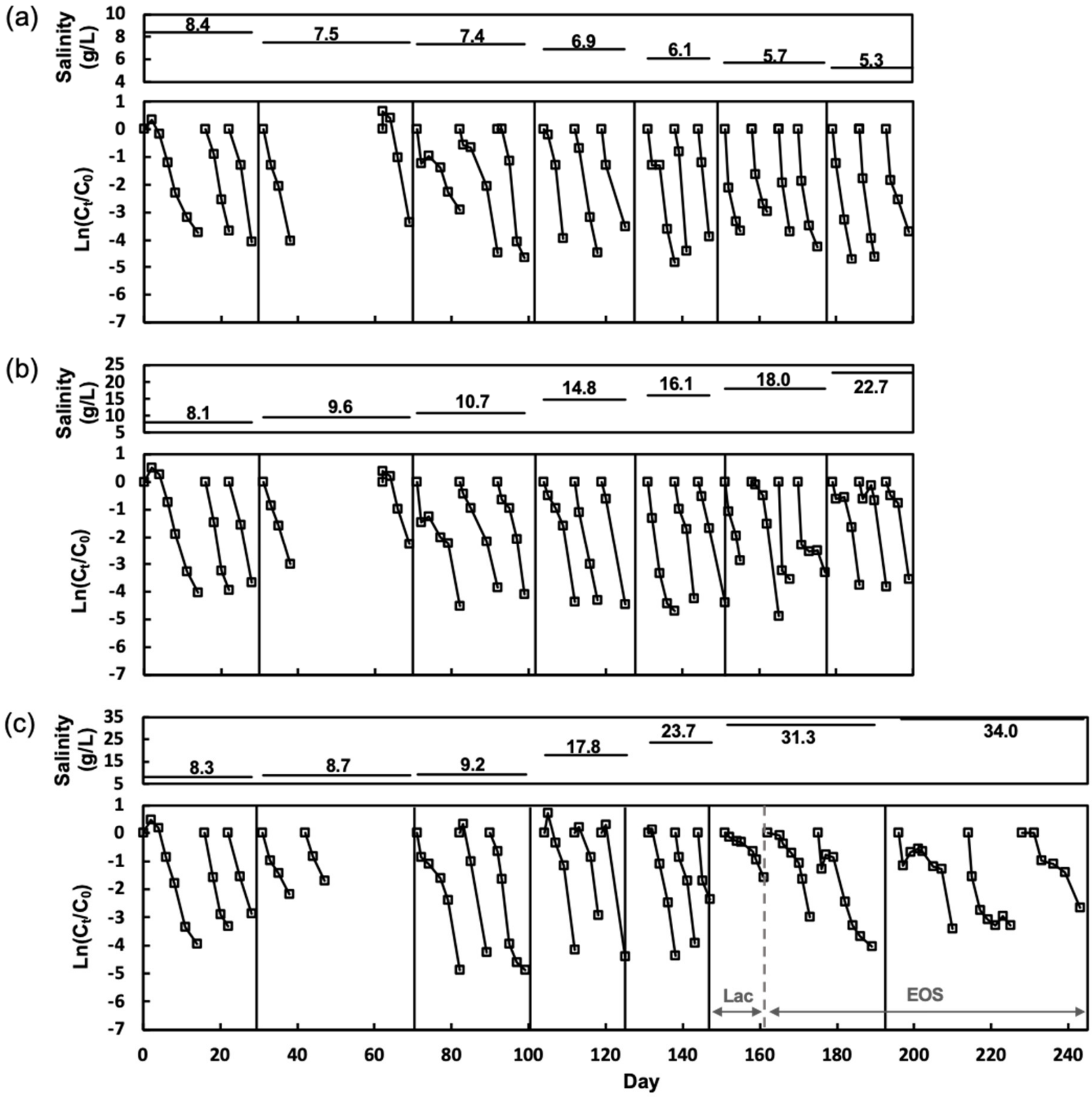

Supplementary Fig. 1 TCE dechlorination in the (a) SD, (b) SI, and (c) SM reactors. TCE was spiked three times at each stage. C0/Ct denotes the chlorine equivalence of chloroethene at time = 0 and time = t at a specific stage. In the SM reactor, lactate served as the electron donor for the first batch at stage T6, and an EOS was used for subsequent batches at stage T6 and all batches at stage T7.

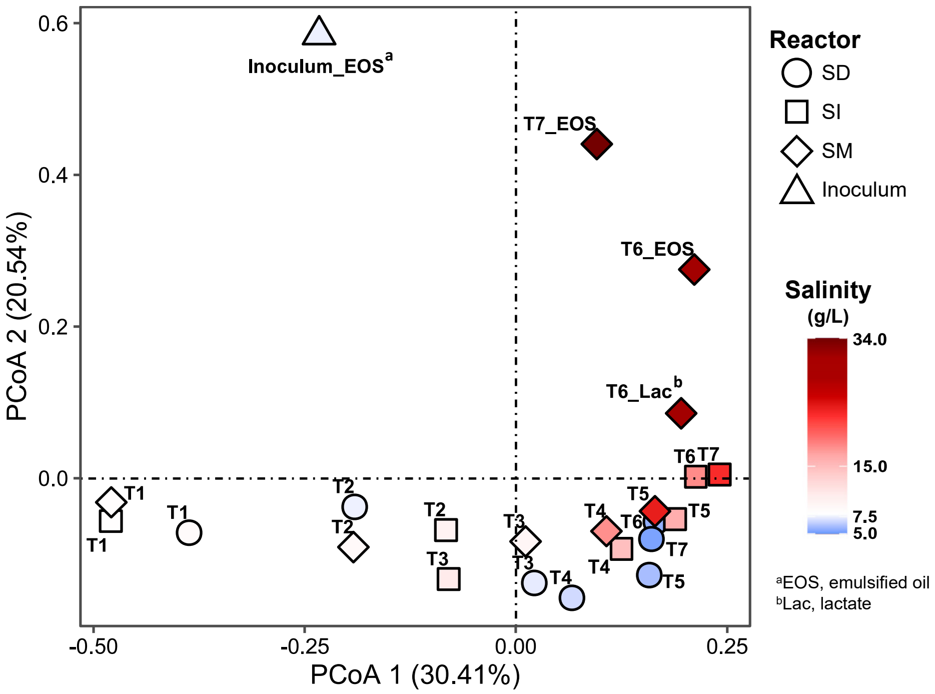

Supplementary Fig. 2 Principal coordinate analysis (PCoA) of microbial communities in the three reactors at different degrees of salinity based on the Bray–Curtis dissimilarity metrics. The text next to the labels represents the stages in the three reactors. The sample DNA underwent full-length 16S PacBio amplicon sequencing. The resulting sequence data were processed and analyzed using QIIME2 (v.2022.2). Each sample was rarefied to a sequencing depth of 24 810 reads to standardize the sequencing effort across samples. Beta diversity was evaluated using the Bray–Curtis dissimilarity metrics, and a PCoA plot was generated to visualize the differences between samples. The first two coordinates explained approximately 50.95% of the observed variation. The microbial community succession proceeded following inoculum with EOS as substrate (Inoculum_EOS), initial stage (T1), and lactate or emulsified oil as the substrate (T2-T7). From stages T2 to T6, the community structures gradually converged without apparent clustering by salinity. The finding indicates that salinity is not the deterministic factor governing community succession. When the substrate was switched from lactate to emulsified oil, a significant shift was observed in the microbial community structure toward the inoculum community, suggesting the substrate effect.

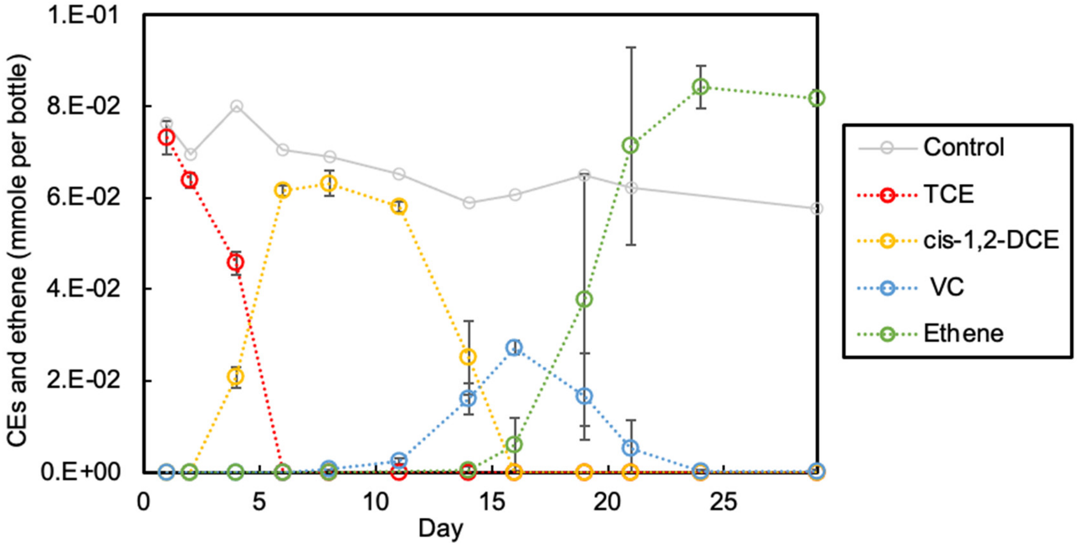

Supplementary Fig. 3 Complete TCE dechlorination at a salinity level of 21 g/L. Batch experiments were conducted in triplicate in 125-mL serum bottles containing 10% (v/v) inoculum from the stepwise increasing salinity (SI) reactor, 45 mL of an anaerobic medium, and 0.05 mL of a vitamin mixture containing vitamin B_{12}. The initial concentration of TCE was approximately 0.07 mmol/bottle. The experiment was conducted at a salinity level of 21 g/L. The data points represent mean values, and the error bars indicate standard deviations (n = 3). These findings support our fed-batch results, indicating the complete dechlorination of TCE to ethene at a salinity level of 21 g/L.

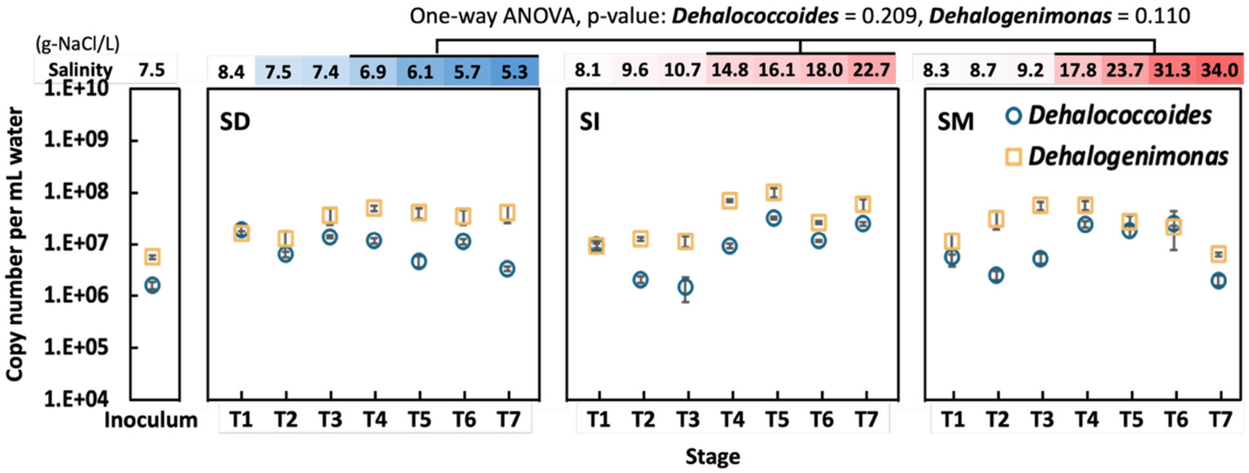

Supplementary Fig. 4 Effects of salinity on *Dehalococcoides* and *Dehalogenimonas* 16S rRNA gene abundance. Changes in the 16S rRNA gene copy numbers per mL of *Dehalococcoides* and *Dehalogenimonas* in response to different salinity levels in three reactors: stepwise decreasing salinity (SD), stepwise increasing salinity (SI), and sharp increase in salinity (SM). Initial inoculum concentration and concentrations at each stage (T1-T7) are shown. One-way ANOVA analysis revealed no significant differences in 16S rRNA gene abundances across salinity levels for both *Dehalococcoides* (p = 0.209) and *Dehalogenimonas* (p = 0.110).

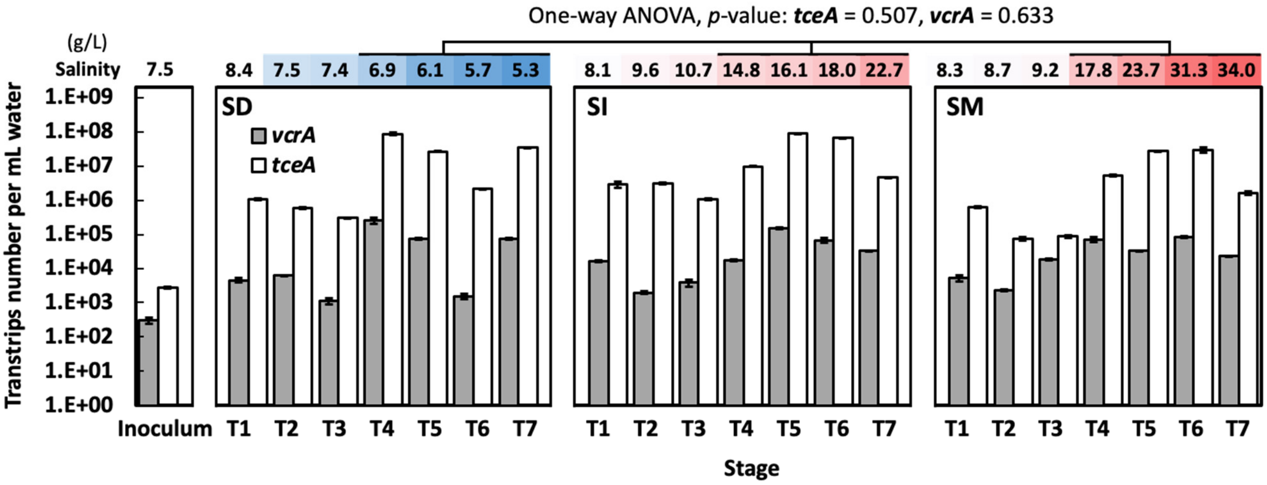

Supplementary Fig. 5 Expression activities of *vcrA* and *tceA* genes at different degrees of salinity. Transcript numbers are expressed as copies per milliliter of water. The data points represent means ± standard deviations (n = 3). Significant differences were determined using one-way ANOVA.

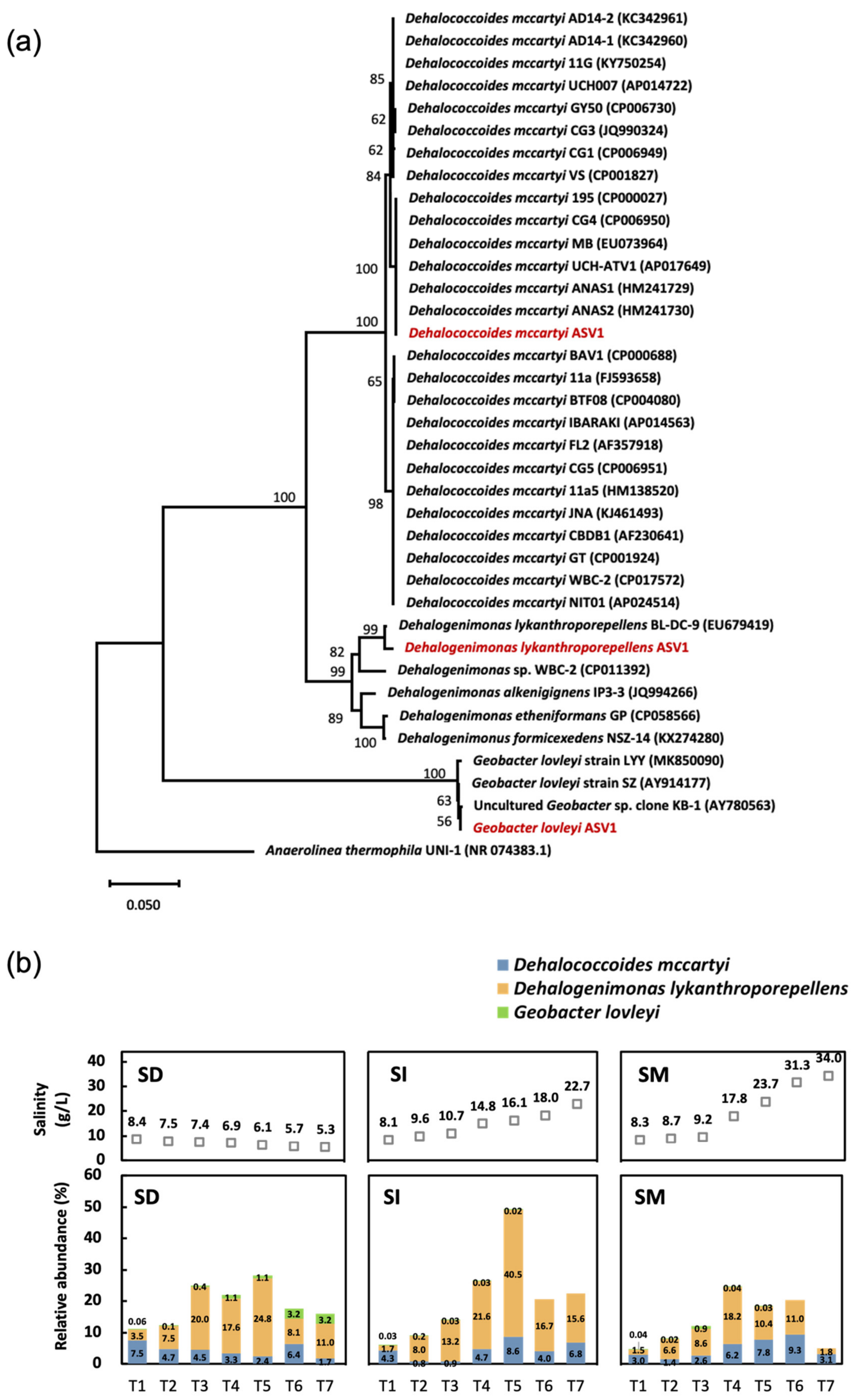
(a)
Dehalococcoides mccartyi AD14-2 (KC342961)
Dehalococcoides mccartyi AD14-1 (KC342960)
Dehalococcoides mccartyi 11G (KY750254)
Dehalococcoides mccartyi UCH007 (AP014722)
Dehalococcoides mccartyi GY50 (CP006730)
Dehalococcoides mccartyi CG3 (JQ990324)
Dehalococcoides mccartyi CG1 (CP006949)
Dehalococcoides mccartyi VS (CP001827)
Dehalococcoides mccartyi 195 (CP000027)
Dehalococcoides mccartyi CG4 (CP006950)
Dehalococcoides mccartyi MB (EU073964)
Dehalococcoides mccartyi UCH-ATV1 (AP017649)
Dehalococcoides mccartyi ANAS1 (HM241729)
Dehalococcoides mccartyi ANAS2 (HM241730)
Dehalococcoides mccartyi ASV1
Dehalococcoides mccartyi BAV1 (CP000688)
Dehalococcoides mccartyi 11a (FJ593658)
Dehalococcoides mccartyi BTF08 (CP004080)
Dehalococcoides mccartyi IBARAKI (AP014563)
Dehalococcoides mccartyi FL2 (AF357918)
Dehalococcoides mccartyi CG5 (CP006951)
Dehalococcoides mccartyi 11a5 (HM138520)
Dehalococcoides mccartyi JNA (KJ461493)
Dehalococcoides mccartyi CBDB1 (AF230641)
Dehalococcoides mccartyi GT (CP001924)
Dehalococcoides mccartyi WBC-2 (CP017572)
Dehalococcoides mccartyi NIT01 (AP024514)
Dehalogenimonas lykanthroporepellens BL-DC-9 (EU679419)
Dehalogenimonas lykanthroporepellens ASV1
Dehalogenimonas sp. WBC-2 (CP011392)
Dehalogenimonas alkenigignens IP3-3 (JQ994266)
Dehalogenimonas etheniformans GP (CP058566)
Dehalogenimonus formicexedens NSZ-14 (KX274280)
Geobacter lovleyi strain LYY (MK850090)
Geobacter lovleyi strain SZ (AY914177)
Uncultured Geobacter sp. clone KB-1 (AY780563)
Geobacter lovleyi ASV1
Anaerolinea thermophila UNI-1 (NR 074383.1)
85
62
62
84
100
100
65
100
98
99
82
99
89
100
100
63
56
0.050
(b)
Dehalococcoides mccartyi
Dehalogenimonas lykanthroporepellens
Geobacter lovleyi
Salinity (g/L)
Relative abundance (%)
SD
SI
SM
8.4 7.5 7.4 6.9 6.1 5.7 5.3
8.1 9.6 10.7 14.8 16.1 18.0 22.7
8.3 8.7 9.2 17.8 23.7 31.3 34.0
T1 T2 T3 T4 T5 T6 T7

Supplementary Fig. 6 Phylogenetic tree and community dynamics of key organohalide-respiring bacteria in TCE-dechlorinating reactors. (a) Phylogenetic tree of the 16S rRNA gene sequences (1400 bp) of *Dehalococcoides*, *Dehalogenimonas* and *Geobacter*, reconstructed using a neighbor-joining algorithm. Sequences were obtained using full-length 16S rRNA gene amplicon sequencing with PacBio technology. The ASVs identified in this study are highlighted in red. Three key organohalide-respiring bacteria were detected: *Dehalogenimonas lykanthroporepellens* ASV1 (99.37% similarity to *Dhg. lykanthroporepellens* BL-DC-9), *Dehalococcoides mccartyi* ASV1 (100% similarity to *D. mccartyi* strain 195, Cornell subgroup), and *Geobacter lovleyi* ASV1. Bootstrap values (1000 replications) are indicated at branch points. The scale bar represents 0.050 substitutions per nucleotide position. (b) Effect of salinity on the relative abundance of *D. mccartyi*, *Dhg. lykanthroporepellens*, and *G. lovleyi* in the three reactors (SD, SI, SM) across different salinity levels. DNA was extracted from sludge samples at each experimental stage (n = 23). Sequencing data were processed using QIIME2 (v.2022.2). Samples were rarefied to 24 810 sequences for standardization. The taxonomic annotation was conducted using the Silva database (release 138) with a 99% similarity cut-off for full-length 16S rRNA gene sequences. Relative abundance was normalized for 16S rRNA gene copy number with the *rrn*DB database (v.5.7) using the QIIME2 plugin q2-gcn-norm. The abundance of *Dhc. mccartyi* fluctuated with changing degrees of salinity in all reactors. The abundance of *Dhg. lykanthroporepellens* increased at lower and higher degrees of salinity without a clear linear relationship. The abundance of *G. lovleyi* tended to decrease as salinity increased. These patterns indicated differential responses to salinity among these key species in TCE-dechlorinating reactors. The top panel displays the degree of salinity (g/L) at each experimental stage (T1–T7), and the bottom panel displays the relative abundance (%) of the three key species.

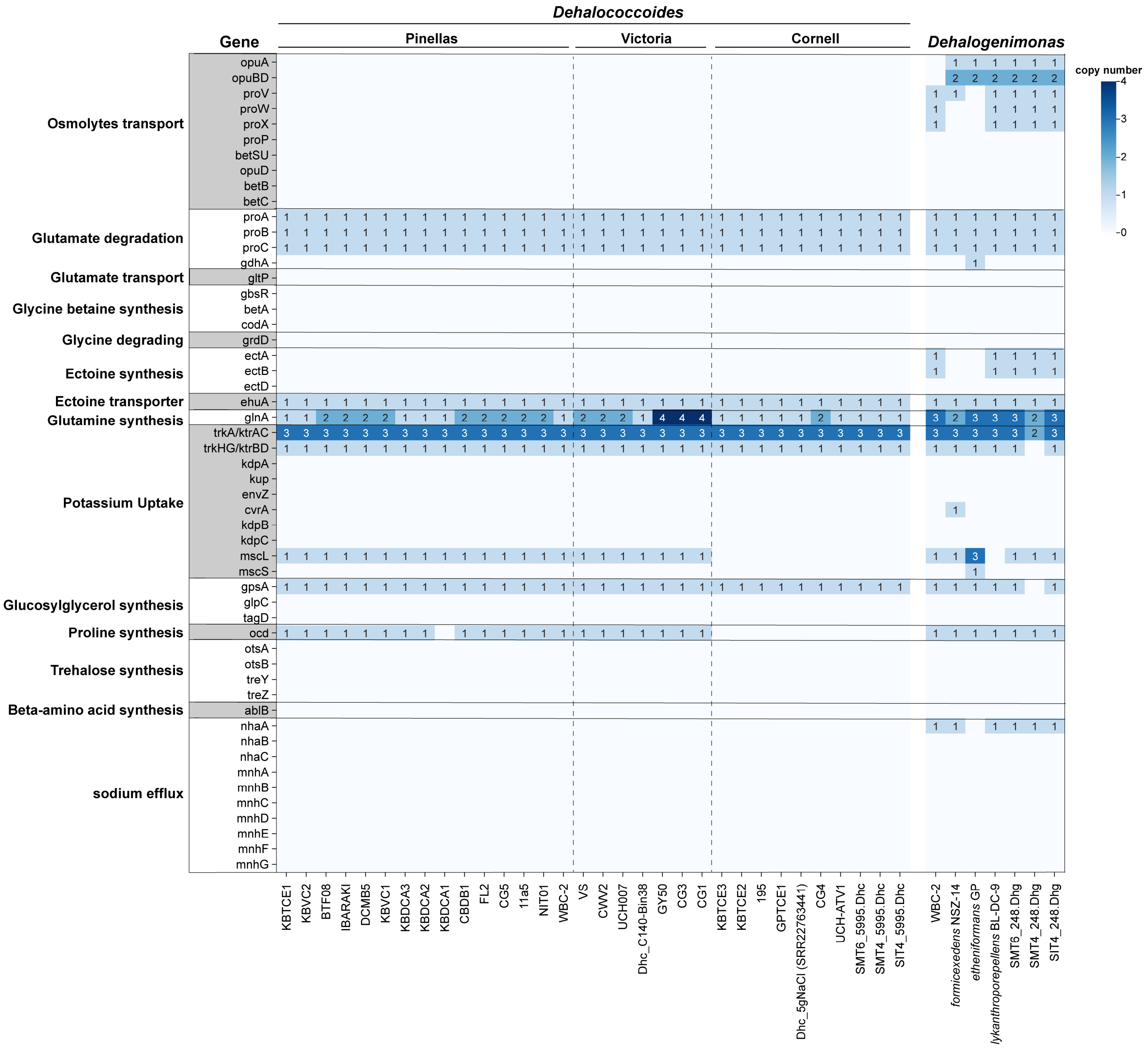

Supplementary Fig. 7 Distribution and number of copies of salt tolerance–related genes in the genomes of *Dehalococcoides* and *Dehalogenimonas*. This heatmap illustrates the number of copies of genes associated with osmolyte transport, compatible solute biosynthesis, and ion homeostasis across different strains of *Dehalococcoides* (divided into Pinellas, Victoria, and Cornell subgroups) and *Dehalogenimonas*. Gene identification and annotation were performed using the EggNOG database and EggNOG-mapper. The color intensity corresponds to the gene copy number, with darker blue indicating higher copy numbers.

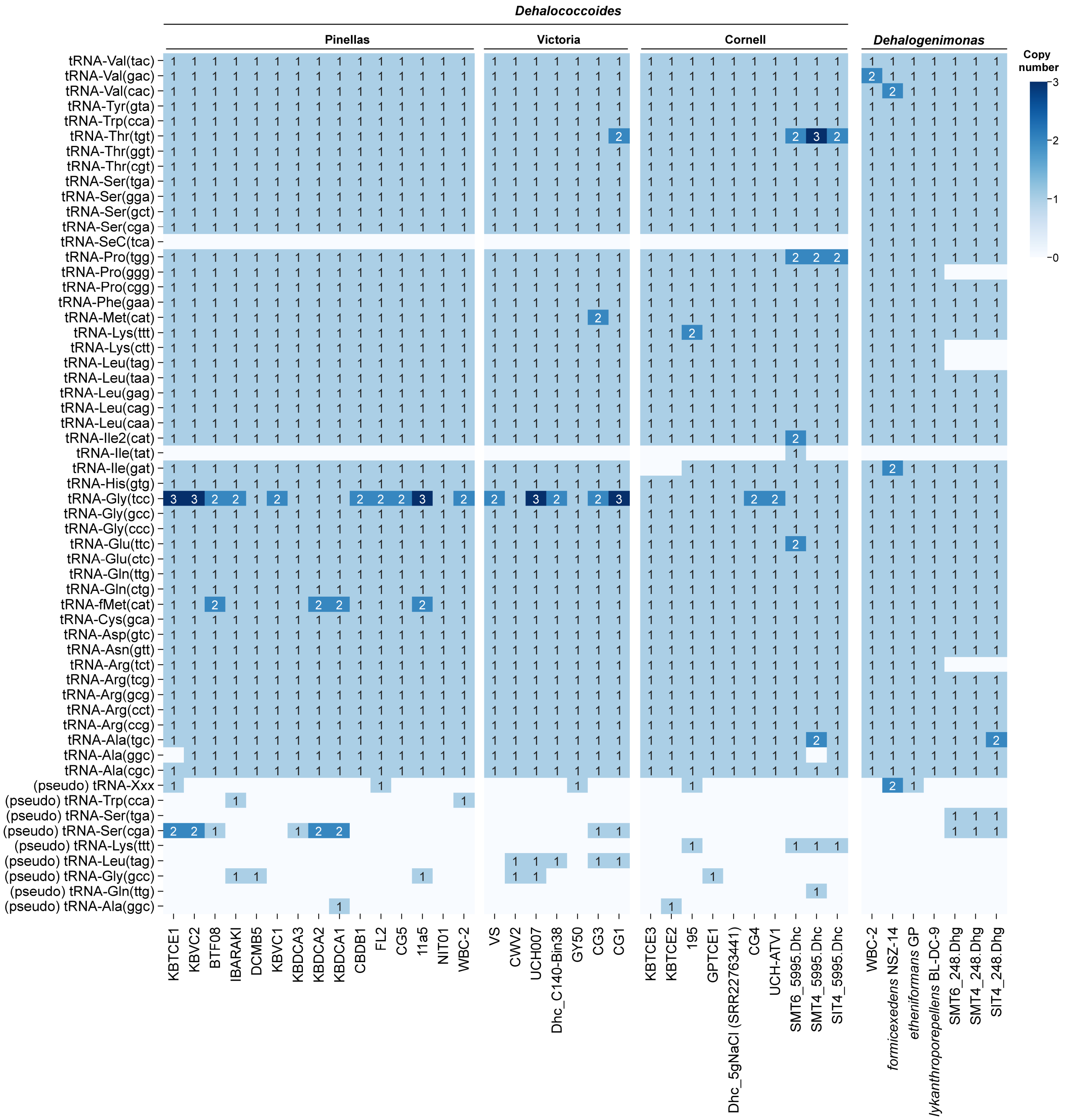

Supplementary Fig. 8 Distribution and copy number of tRNA genes in the genomes of *Dehalococcoides* and *Dehalogenimonas*. The heatmap illustrates the diversity and number of copies of tRNA genes, categorized by amino acid specificity and anticodon sequence, in various strains of *Dehalococcoides* (subdivided into Pinellas, Victoria, and Cornell subgroups) and *Dehalogenimonas*. tRNA annotation was performed using Bakta (v.1.8.2) on metagenome-assembled and reference genomes. Each row represents a specific tRNA type, and each column represents an individual bacterial strain. The color intensity indicates the copy number of each tRNA gene, with darker blue representing higher copy numbers. Pseudo-tRNAs are included at the bottom of the chart. This comprehensive tRNA profiling offers insights into the translational capacity and potential codon usage bias of these organohalide-respiring bacteria.

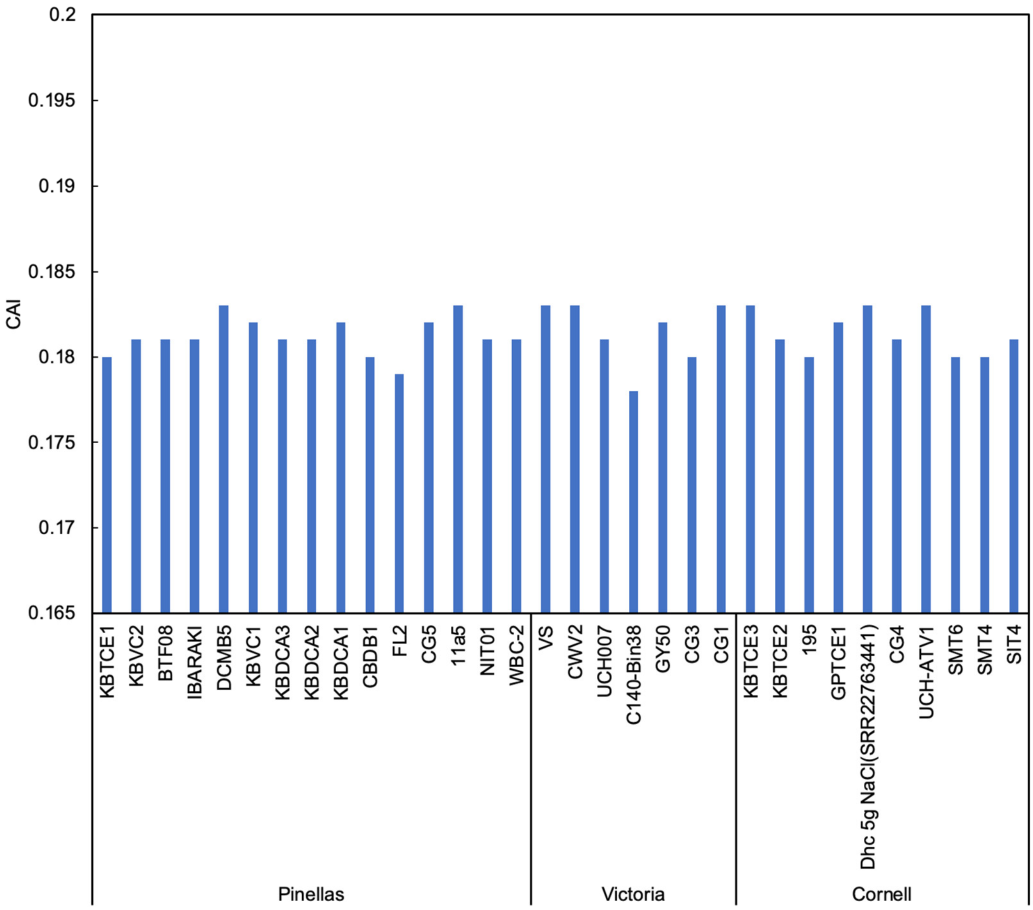

Supplementary Fig. 9 Distribution of codon adaptation index (CAI_{ave}) values across different strains of *Dehalococcoides*. The bar chart illustrates the CAI_{ave} values corresponding to various *Dehalococcoides* genomes, divided into Pinellas, Victoria, and Cornell subgroups. CAI_{ave} represents the mean codon adaptation index of each genome, calculated using CodonW (v.1.4.2) on all coding sequences.

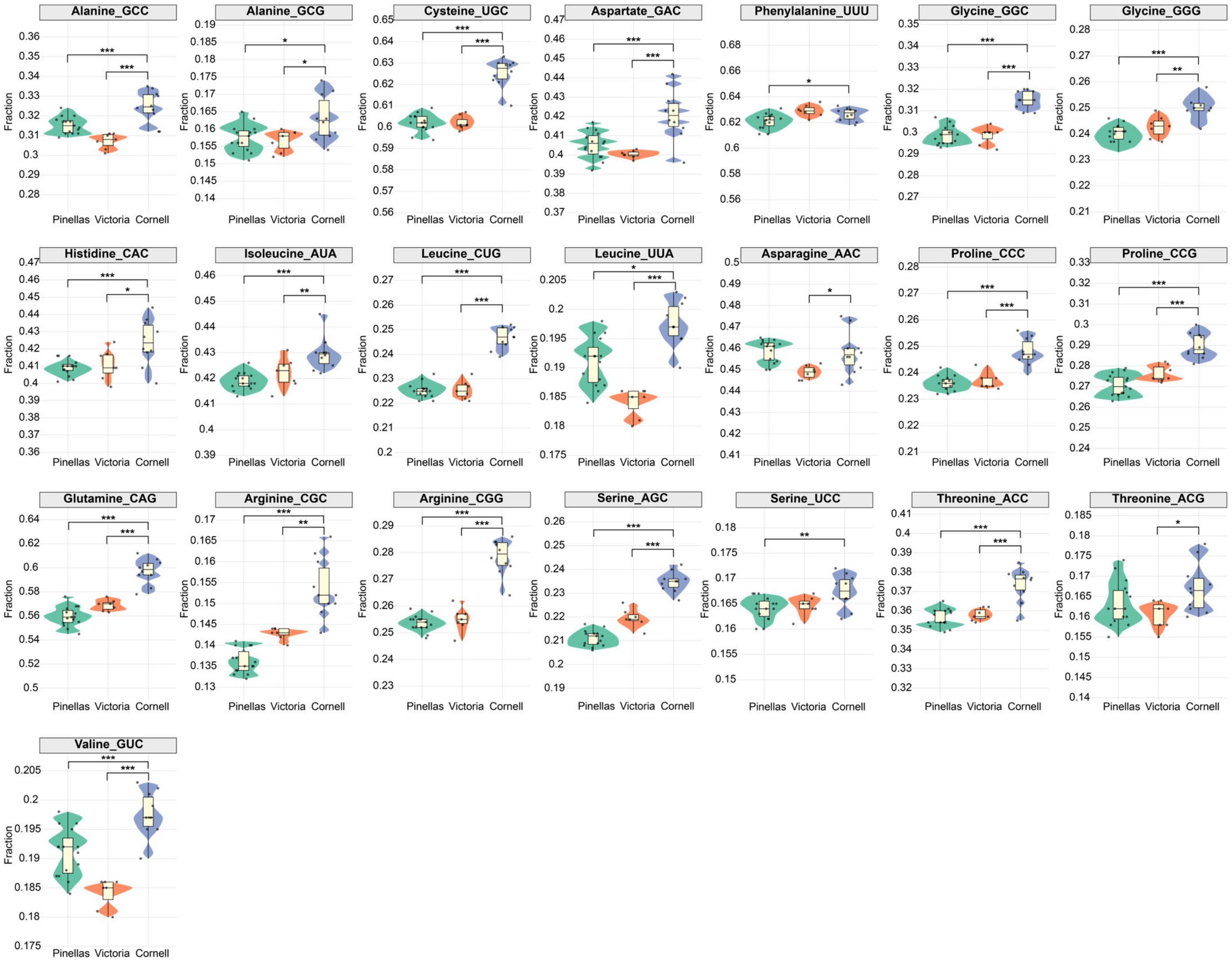

Supplementary Fig. 10 Distinct codon usage preferences in different subgroups of *Dehalococcoides*. This figure illustrates the codon usage preferences for 22 codons corresponding to 15 amino acids across the three subgroups of *Dehalococcoides* (i.e., Pinellas, Victoria, and Cornell). The codon fraction represents the proportion of a specific codon's usage among its synonymous codons for each amino acid. Codon usage was analyzed using the CUSP program of EMBOSS. The Cornell subgroup exhibited significantly higher usage of 22 preferred codons for 15 amino acids compared with the Pinellas and Victoria subgroups. By contrast, the Pinellas and Victoria subgroups exhibited significantly higher usage of 23 and 21 preferred codons, respectively, for 15 amino acids compared with the Cornell subgroup. Significant differences in codon usage were determined using one-way ANOVA followed by Tukey's post hoc test. Statistical significance is indicated by asterisks: $^{*}p < 0.05$, $^{**}p < 0.01$, and $^{***}p < 0.001$.

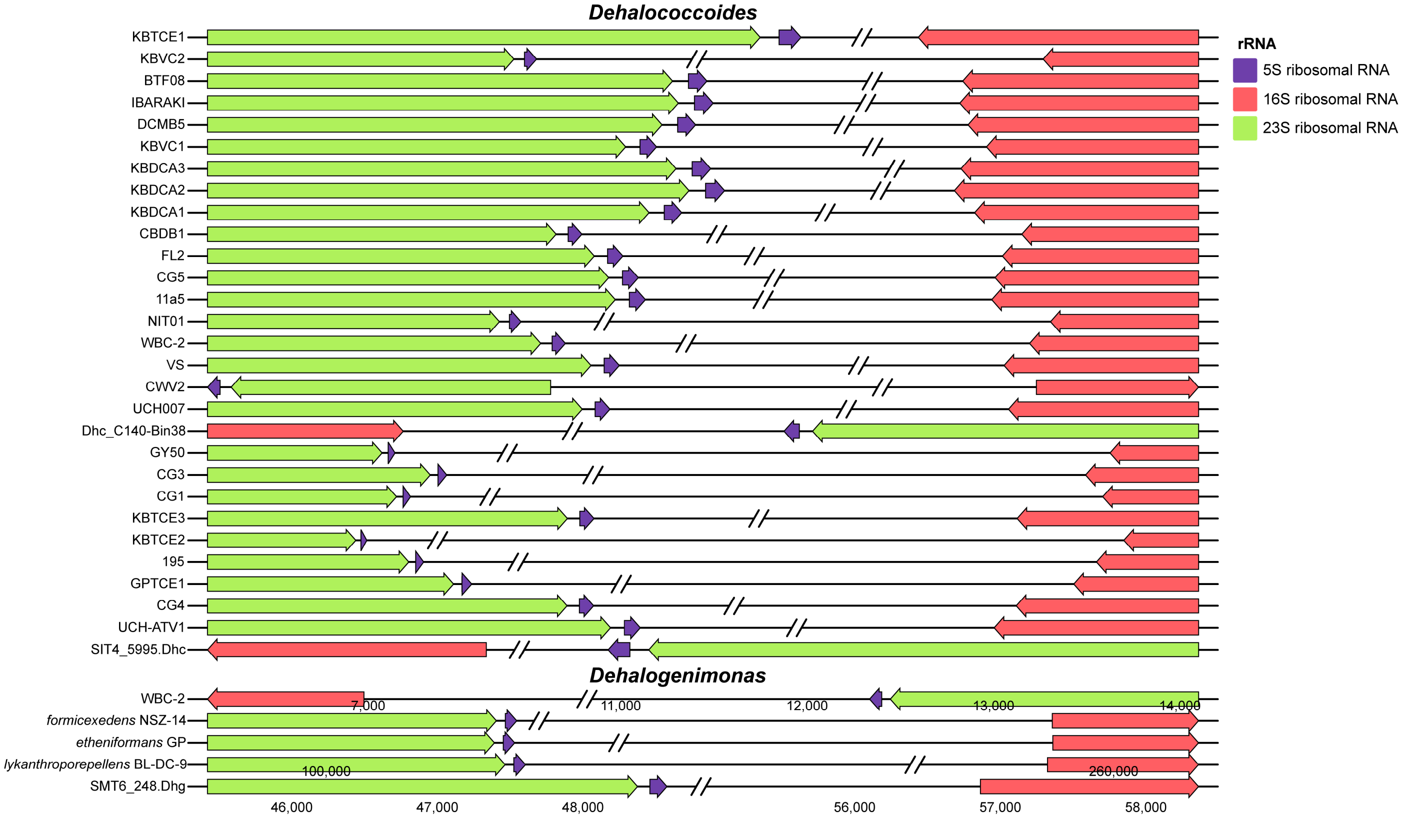

Supplementary Fig. 11 Schematic representation of rRNA gene locations across multiple strains of *Dehalococcoides* and *Dehalogenimonas*. Colored bars represent different rRNA genes: purple for 5S rRNA, red for 16S rRNA, and green for 23S rRNA. Double forward slashes (//) in the diagram represent breaks in the continuous sequence to show distant genomic regions. Numbers indicate genomic positions. Strain names are listed on the left. The diagram shows that the 5S, 16S, and 23S rRNA genes are not co-localized inside the same operon in these genera.

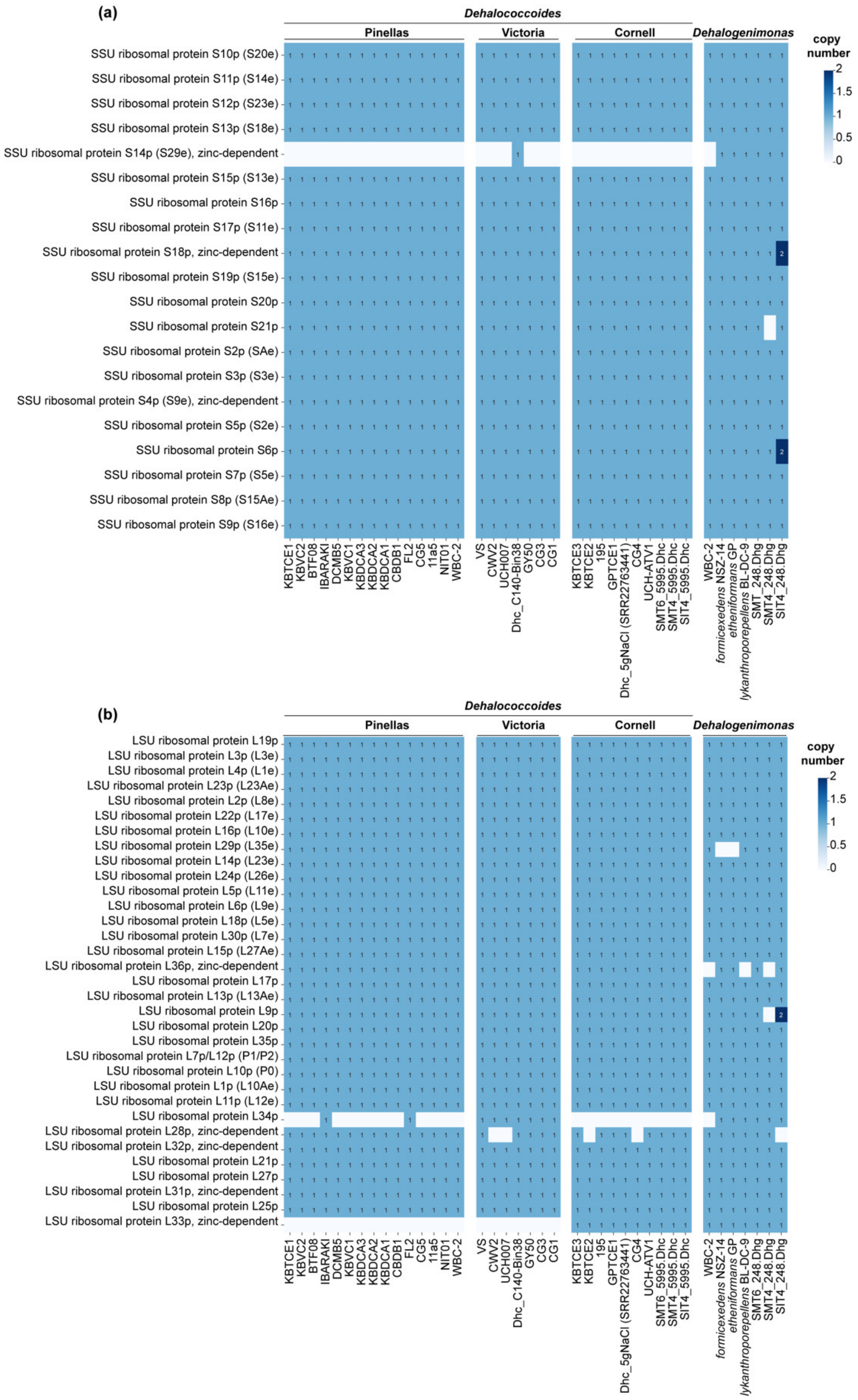

Supplementary Fig. 12 Distribution and copy number of ribosomal protein genes in the genomes of *Dehalococcoides* and *Dehalogenimonas*. These heatmaps indicate the presence and copy numbers of (a) SSU and (b) LSU ribosomal protein genes across the *Dehalococcoides* subgroups (Pinellas, Victoria, and Cornell) and *Dehalogenimonas*. Genomic analysis was conducted using Bakta (v.1.8.2) on MAGs and reference genomes. This analysis identified 20 SSU and 33 LSU ribosomal protein genes typically present as single copies. The color intensity corresponds to the number of copies, with darker blue indicating higher copy numbers and white indicating no copies.

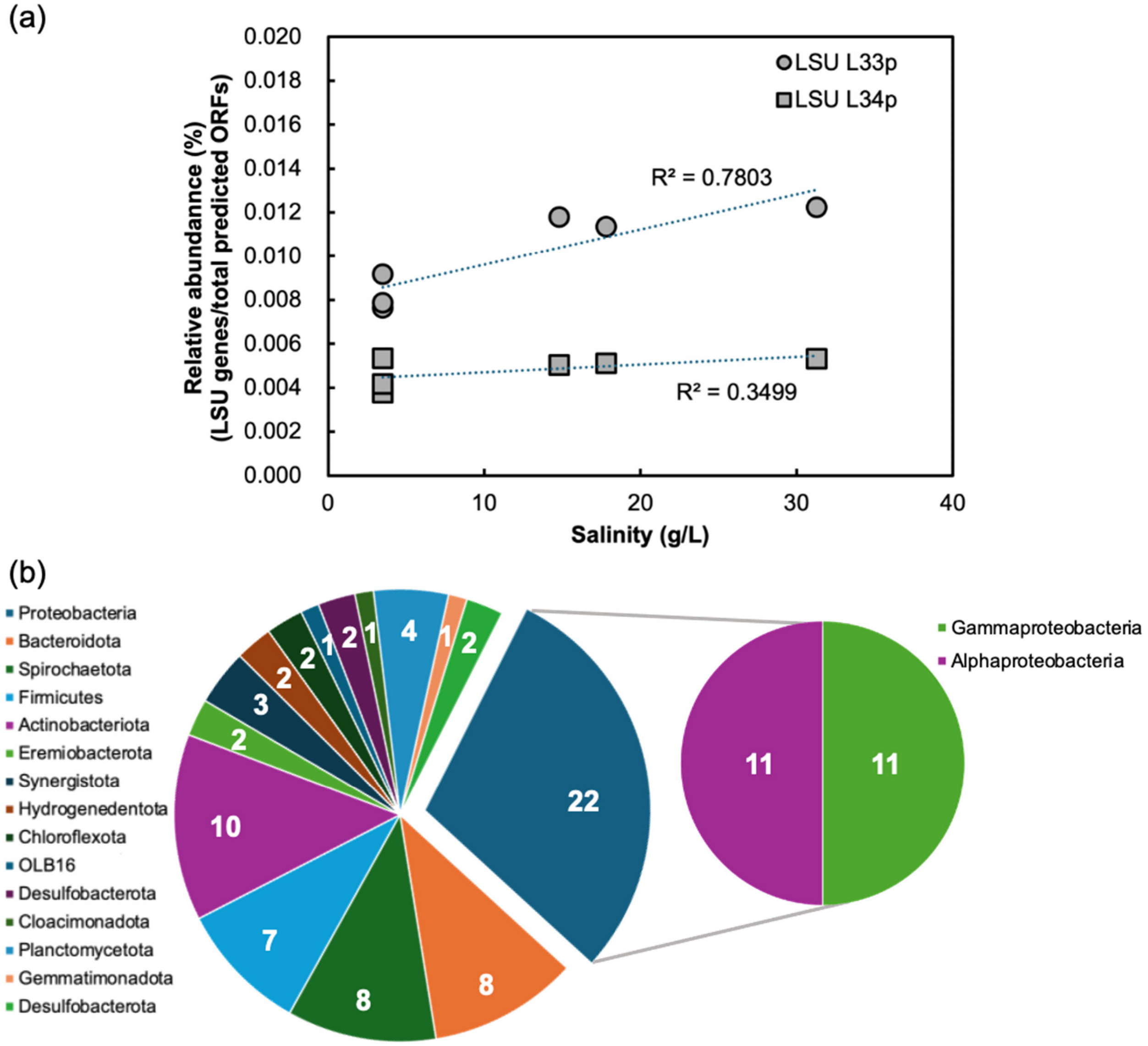

Supplementary Fig. 13 Correlation between salinity and LSU ribosomal protein gene abundance, and taxonomic distribution of L33p-containing MAGs. (a) Relative abundance of LSU L33p and L34p genes at different salinity levels. The y-axis displays the relative abundance, defined as the ratio of gene copy number to total predicted open reading frames in each sample. A significant positive correlation was observed between salinity and L33p gene abundance ($\beta = 2\times10^{-4}$, $R^2 = 0.78$), while L34p showed a weaker correlation ($\beta = 3\times10^{-5}$, $R^2 = 0.35$). (b) Taxonomic distribution of metagenome-assembled genomes (MAGs) containing the LSU L33p gene from the sixth stage of the SM reactor. MAGs were filtered for quality (>50% completeness and <10% contamination). The pie chart illustrates the phylum-level distribution, with the number of MAGs indicated for each taxon. The inset expands on the *Proteobacteria* phylum, showing class-level distribution. The diverse taxonomic representation of L33p-containing MAGs suggests a widespread occurrence of this gene across various bacterial lineages in this salinity environment.

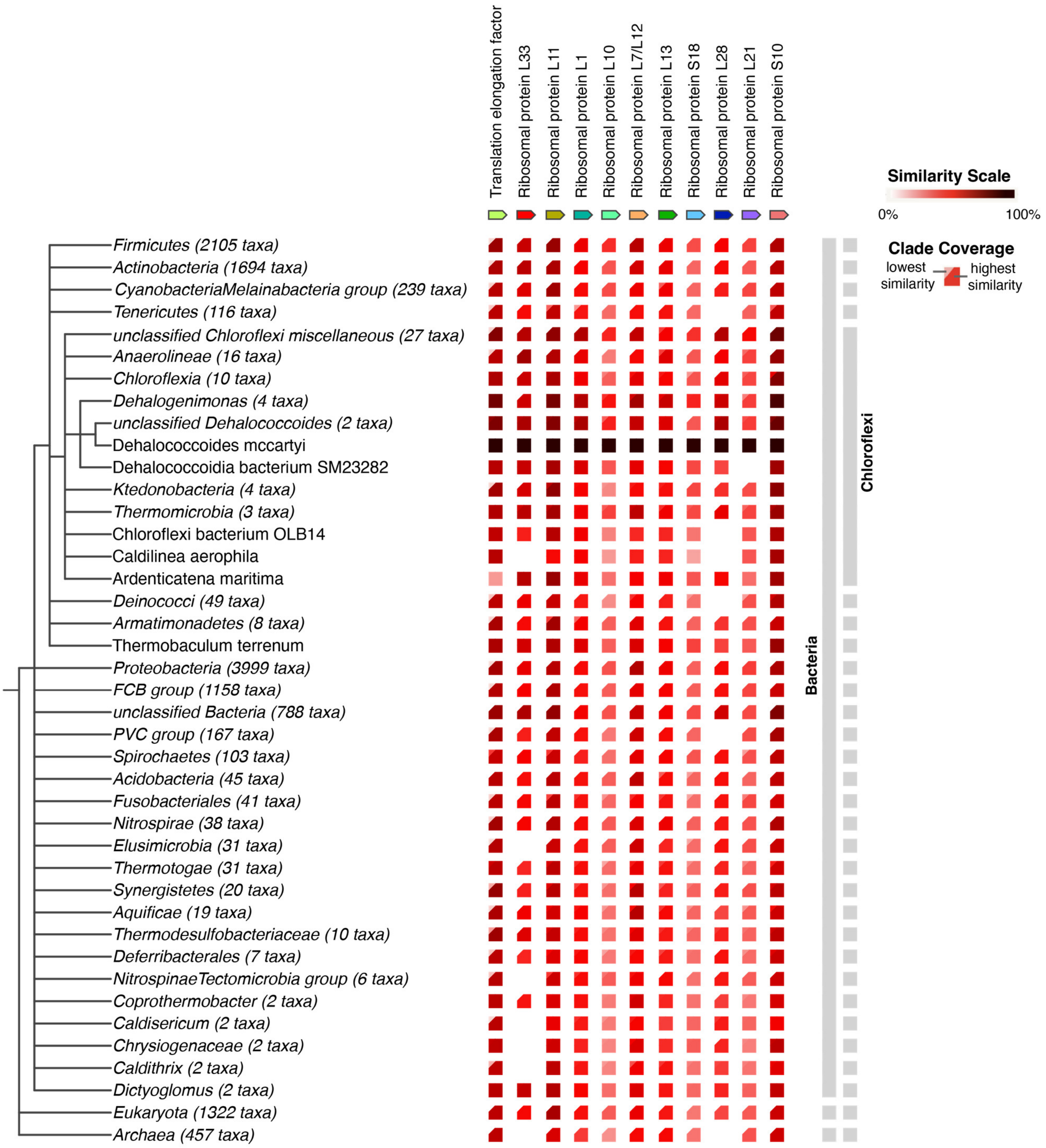

Supplementary Fig. 14 Gene co-occurrence analysis revealing the distribution pattern of ribosomal protein L33 across microbial taxa. Gene co-occurrence profiles for ribosomal protein L33 and associated proteins across diverse microbial taxa, generated using STRING database (v12.0). The phylogenetic tree on the left represents the taxonomic relationships among the analyzed groups. Colored squares indicate the presence and similarity of each gene (columns) in different taxa (rows), with darker shades representing higher similarity to the query sequence. Co-occurrence analysis revealed an association between ribosomal protein L33 and several ribosomal proteins, including L11, L1, L10, L7/L12, L13, S18, L28, L21, and S10, suggesting potential functional relationships., suggesting a potential functional relationship. The analysis highlights the variable distribution of L33 across microbial taxa. Its uneven distribution throughout microbial taxa suggests the varied evolutionary histories of ribosomal protein L33.
